# Excessive Flexibility? Recurrent Neural Networks Can Accommodate Individual Differences in Reinforcement Learning Through In-Context Adaptation

**DOI:** 10.1007/s42113-025-00254-8

**Published:** 2025-07-18

**Authors:** Kentaro Katahira

**Affiliations:** Human Informatics and Interaction Research Institute, National Institute of Advanced Industrial Science and Technology (AIST), Tsukuba, Japan

**Keywords:** Recurrent neural networks, Cognitive computational modeling, Reinforcement learning, Individual differences

## Abstract

Cognitive and computational modeling has been used as a method to understand the processes underlying behavior in humans and other animals. A common approach in this field involves the use of theoretically constructed cognitive models, such as reinforcement learning models. However, human and animal decision-making often deviates from the predictions of these theoretical models. To capture characteristics that these cognitive models fail to account for, recurrent neural networks (RNNs) have been increasingly used to model choice behavior involving reinforcement learning. RNNs can capture how choice probabilities change depending on past experience. In this work, we demonstrate that RNNs can improve future choice predictions by capturing individual differences on the basis of past behavior, even when a single model is fit across the entire population. We refer to this capacity as the individual difference tracking (IDT) property. While the IDT property might be useful for prediction, it may introduce excessive flexibility when RNNs are used as benchmarks for predictive accuracy. We investigate the nature of the IDT property through simulation studies and examine how it affects the interpretation of predictive accuracy when RNNs are used as benchmarks for cognitive models. We also present examples using real-world data. Through these analyses, we discuss practical considerations and limitations in using RNNs as benchmarks for cognitive models.

**Supplementary Information:**

The online version contains supplementary material available at 10.1007/s42113-025-00254-8.

## Introduction

Cognitive computational modeling, in which models representing cognitive and computational processes are applied to human and animal behavioral data to infer underlying processes, is becoming an essential method in behavioral analysis (Daw, 2011; Wilson & Collins, 2019). Reinforcement learning (RL) models are commonly employed to examine reward learning in humans and animals (Yechiam et al., 2005; Corrado & Doya, 2007; Daw, 2011; Wilson & Collins, 2019). However, such models are susceptible to misspecification, where the true underlying processes may not be captured, leading to erroneous interpretations (Nassar & Gold, 2013; Nassar & Frank, 2016). Specifically, model misspecification can lead to bias in the parameter estimates because the components not included in the model might be compensated for by other components (Nassar & Gold, 2013; Katahira, 2018; Toyama et al., 2019a; Sugawara & Katahira, 2021). Furthermore, in model-based functional magnetic resonance imaging (fMRI) analyses—where computational models are used to relate behavior to neural activity—model misspecification has led to spurious group differences (Katahira & Toyama, 2021). Thus, researchers must develop models that represent actual underlying processes and fit behavioral data. Nevertheless, in the analysis of real-world experimental data, where the true model is unknown, determining whether a model fit is sufficient is challenging. This difficulty arises because behavioral choices inherently involve stochastic components that cannot be fully explained by any model, making it difficult to determine which aspects of variability are due to cognitive processes that should be modeled and which are more appropriately treated as noise.

To address this issue, the application of highly flexible artificial neural networks (ANNs) has gained attention in recent years. When ANNs are used as benchmarks, researchers can compare their predictive performance with that of computational cognitive models to assess whether they adequately capture the variability in data and whether any important cognitive components are missing (Dezfouli et al., 2019; Song et al., 2021; Fintz et al., 2022; Eckstein et al., 2024). If the predictive performance of a cognitive model is clearly inferior to that of an ANN benchmark, it suggests that the model lacks essential elements. In such cases, researchers may iteratively refine the cognitive model by incorporating additional components and reevaluating its performance. The use of ANN benchmarks thus provides a means of determining how much further model refinement may be needed.

In the context of reward learning in humans and other animals, recurrent neural networks (RNNs), a type of ANN for learning sequential data, are often employed (Dezfouli et al., 2019; Song et al., 2021; Fintz et al., 2022; Ji-An et al., 2023; Ger et al., 2024a; Rmus et al., 2024; Eckstein et al., 2024). Among various RNN architectures, long short-term memory (LSTM) (Hochreiter, 1997) and gated recurrent units (GRUs) (Cho, 2014) are particularly prevalent. These architectures are capable of capturing long-term influences from past events, offering more flexible learning than theory-based cognitive models such as RL models do (Dezfouli et al., 2019). This flexibility enables RNNs to better model the complex structure of behavior.

On the other hand, RNNs typically have a much larger number of parameters than theory-based cognitive models do, which often requires a relatively large amount of data for effective training. As a result, it is generally difficult to fit RNNs individually for each participant. Thus, researchers typically pool data across participants and train a single RNN model based on the entire dataset.

In general, when the amount of data is insufficient relative to the number of parameters, a model may overfit to noise or incidental structures unrelated to the true data-generating process, resulting in poor generalizability. In statistical models such as cognitive models, information criteria such as Akaike’s information criterion (AIC; Akaike, 1974) and the Bayesian information criterion (BIC; Schwarz et al., 1978) or model evidence (i.e., marginal likelihood) are commonly used to penalize excess model complexity (Daw, 2011). However, in ANNs such as RNNs, the number of parameters (i.e., weights) does not correspond directly to model flexibility, and information criteria applicable to RNNs have not yet been established.

As a practical solution, model performance for RNNs is typically evaluated based on predictive accuracy on held-out test data (Dezfouli et al., 2019; Song et al., 2021; Eckstein et al., 2024). In RL tasks, however, data from successive trials are often dependent on the sequential structure of the task. When participants are exposed to only one pair of stimuli, it is difficult to split the data within a session into training and test sets because subsequent responses may be influenced by earlier trials. In such cases, a common strategy is to split the dataset by participants—using some participants for training and others for testing (see Supplementary Fig. S1B for a schematic illustration). Under this approach, participant-specific models fitted to training data are unsuitable for evaluating individual performance, as those individuals are absent from the test set. Therefore, RL models must be fitted using a single set of parameters shared across all participants.

However, when participants complete multiple independent sessions (e.g., with different stimulus pairs), training and test samples can be split at the session level (see Supplementary Fig. S1A for a schematic illustration). In such cases, it becomes possible to fit RL models individually for each participant using one session and evaluate the predictive accuracy with the other (Song et al., 2021). Nonetheless, this type of design is still relatively rare.

Given these constraints, when comparing RNNs with theory-based cognitive models such as RL models, it is common practice to also fit RL models using a single set of parameters shared across participants (Dezfouli et al., 2019; Fintz et al., 2022; Eckstein et al., 2024). This approach is generally referred to as a fixed-effect model in the statistical modeling literature. In this study, we refer to it as a “common fit” to emphasize that the same parameter set is applied to all individuals. From this perspective, comparing RL models and RNNs in terms of model fit and predictive accuracy can be considered fair, as both approaches rely on a single group-level parameterization.

However, we demonstrate that even a single RNN might learn individual differences from behavior during early trials and use this information to predict subsequent behavior. This occurs through what is known as in-context adaptation or in-context learning, a property of RNNs whereby the internal state of the network is dynamically updated based on the sequence of observed inputs—allowing the model to adjust its behavior on a per-individual basis without explicitly estimating separate parameters for each individual. We refer to this ability as the *individual difference tracking* (IDT) property of RNNs. We suggest that, owing to the IDT property, RNNs might overestimate the prediction accuracy when used as a benchmark against cognitive models that assume shared parameters across individuals. We also discuss the implications of these properties for cognitive and computational modeling.

Notably, this paper is not the first to mention the IDT property of RNNs: Dezfouli et al. (2019) noted this possibility in the last paragraph of their Discussion section. The novel contributions of this paper are as follows. First, we illustrate the IDT property of RNNs using data generated from numerical simulations based on simple RL models. We then examine the extent to which RNNs can express individual differences through IDT across various scenarios involving different underlying generative models. These simulations demonstrate how the presence or absence of IDT affects the interpretability of RNNs as predictive benchmarks for cognitive models. The results show that RNNs do not always track individual differences accurately and, in many cases, perform worse than cognitive models that are individually fitted to data do. We also propose a method to quantify the degree of IDT in a trained RNN, which we refer to as the *on-policy IDT check* and investigate factors that suppress IDT, such as early stopping and architectural constraints. In addition to synthetic data simulations, we present empirical demonstrations using real-world behavioral datasets to illustrate how RNNs can be used as benchmarks for cognitive models while taking IDT into account. Finally, we discuss how RNNs should be used as benchmarks for evaluating cognitive models, considering the presence of IDT and its limitations, and suggest directions for future research.

## Simulation Settings

In this paper, we first discuss the properties of RNNs trained using behavioral data generated from simple RL models, where the true underlying process is known. This setting allows us to evaluate how well RNNs capture the structure of the data, including individual differences. We simulated the choice behavior of 100 agents (virtual subjects) engaged in a two-armed bandit task using various RL models with systematically varied parameters such as the learning rate. Specifically, to generate the synthetic data, we used variants of *Q*-learning models^1^. Among the variants, we focus in particular on the forgetting *Q*-learning (FQ-learning) model (Ito & Doya, 2009). The behavior of this model is theoretically guaranteed to be replicable even with the simplest form of RNN (a linear RNN with a single RNN cell; see Appendix C). More complex RNN architectures (e.g., vanilla RNN, LSTM, and GRU) are likely to exhibit similar capabilities, allowing us to eliminate the influence of model misspecification and focus on the effects of individual differences.

In *Q*-learning models, the *Q*-value or action value, $Q_t(a_t)$, for a chosen option $a_t \in \{A, B\}$ at trial *t* is updated as

1 $$\begin{aligned} Q_{t+1}(a_t) = Q_t(a_t) + \alpha (r_t - Q_t(a_t)), \end{aligned}$$

where $\alpha \in [0,1]$ is the learning rate, which determines the extent to which the prediction error affects the updated value, and where $r_t \in \{0, 1\}$ is the reward received at trial *t*.

In standard *Q*-learning, the *Q*-value of the unchosen option remains unchanged. In the forgetting variants of *Q*-learning, the *Q*-value for the unchosen option $\bar{a}_t$ is assumed to decay as follows:

2 $$\begin{aligned} Q_{t+1}(\bar{a}_t) = (1 - \alpha _F) Q_t(\bar{a}_t), \end{aligned}$$

where $\alpha _F$ is the forgetting rate, which determines the rate at which the value of the unchosen option decays. In FQ-learning, $\alpha _F$ is set as $\alpha _F = \alpha$ (the forgetting rate is identical to the learning rate). The standard *Q*-learning model corresponds to the setting where $\alpha _F = 0$.

The choice probability (for option A) is determined by the softmax function:

3 $$\begin{aligned} P(a_t = A) = \frac{1}{1+\exp \left( - \beta ( Q_t(A) - Q_t(B) ) \right) }. \end{aligned}$$

where $\beta$ is the inverse temperature, which governs the sensitivity of the choice probability to differences in option values. A larger $\beta$ results in a more sensitive change in the choice probability. In the FQ-learning model, individual differences are represented as the differences in the learning rate $\alpha$ and the inverse temperature $\beta$. Notably, in FQ-learning, $\alpha$ also determines the strength of forgetting (i.e., the decay rate) for the values of the unchosen options.

The agent simulated via RL models engages in a two-armed bandit task (probabilistic reversal learning task), where it receives rewards based on the reward probabilities associated with each option; the reward probabilities switch every 50 trials. Each agent completes two sessions of the task, with each session consisting of 200 trials. One session is used to generate training data for the RNN and RL models, and the other is used as test data to evaluate the predictive accuracy of the models. For further details about the task, see Appendix A.1. In the simulation, we assumed 100 subjects (agents), each modeled using the RL models.

For RNN model training and evaluation, the training data from all 100 subjects were pooled to train a single model. The predictive performance was then evaluated based on the test data by computing the normalized log-likelihood for each subject (see Appendix A.4 and A.6 for the details).

For RL model fitting, we applied both the common fit approach, where a single parameter set is estimated by pooling the training data across all 100 subjects, similar to the RNN, and the individual fit approach, where parameters are estimated separately for each subject. Details of the model fitting and evaluation procedures are provided in Appendix A.3 and A.6. As noted in the Introduction, the common fit approach is often applied when the subjects in the training and test datasets differ. In our simulation settings, however, the same subjects (i.e., agents with identical parameter values) were included in both the training and test datasets. This design is expected to be comparable to real-world scenarios, as long as the parameter distributions across subjects in empirical data are not substantially different between the training and test sets.

**Fig. 1:**
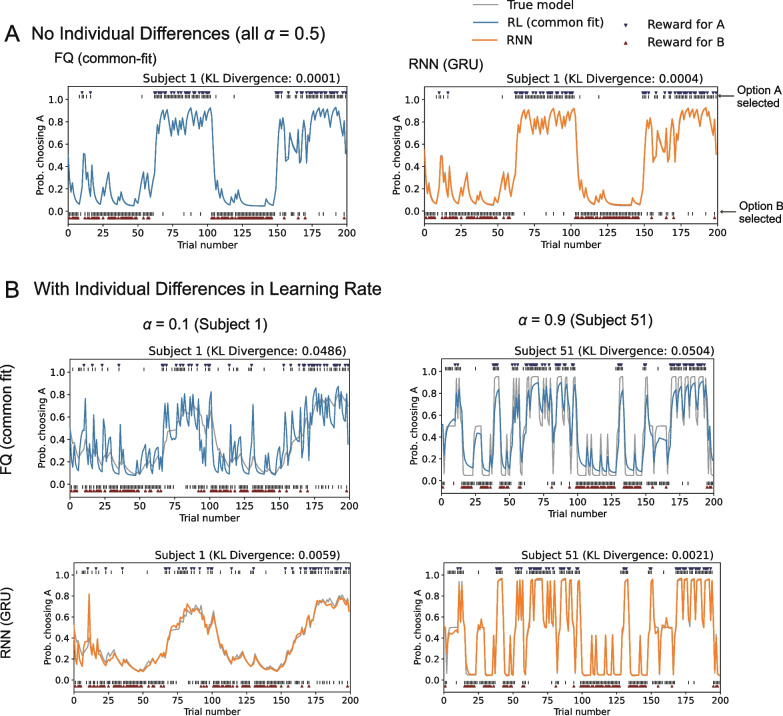
Illustration of the individual difference tracking (IDT) property of an RNN. The plots show the trajectories of choice probabilities output by a common-fit FQ-learning model (shown in blue lines) and an RNN (here, a GRU; shown in orange lines) trained based on data simulated using the FQ-learning model (shown in gray lines) in a two-armed bandit task. **A** Case without individual differences, where all the subjects share a common learning rate ($\alpha = 0.5$). **B** Case with individual differences, where half of the subjects (Subjects 1 to 50) have a low learning rate ($\alpha = 0.1$) and the other half (Subjects 51 to 100) have a high learning rate ($\alpha = 0.9$). The upper panels show the results of fitting an FQ model with common parameters (blue), and the lower panels show the results from the RNN (GRU). KL: Kullback–Leibler divergence between the true and predicted choice probabilities. A value of zero indicates perfect agreement between the model’s prediction and the true choice probability

## Illustration of the IDT Properties of an RNN

We begin by considering a case where the ground-truth model is an FQ-learning model, and both an FQ-learning model with common parameters and an RNN are fitted to the simulated data. In the following analyses, we primarily use a GRU-based architecture for the RNN. Unless otherwise noted, the term “RNN” refers to an RNN implemented with a GRU (for details on the RNN architecture, see Appendix A.4). The simulated data were pooled across subjects, and each model (RNN and FQ-learning) was trained to estimate a single set of parameters using the entire dataset. Choice predictions were generated using off-policy simulation, meaning that each model predicted the probability of the choice at trial *t* based on the history of choices and rewards up to trial $t-1$, without actually selecting actions.

Figure 1A shows the choice probabilities produced by the RNN and the common-fit FQ-learning model when all the subjects shared the same (ground-truth) learning rate ($\alpha = 0.5$), implying no individual differences. The gray lines represent the true choice probabilities for option A generated by the FQ-learning model (almost completely overlapped with the colored lines in this case). In this case, the choice probabilities predicted by the common-fit FQ model (left panel, blue line) closely match the true choice probabilities generated by the ground-truth FQ model (gray line). This result is expected, as the fitted model structure, including the absence of individual differences, perfectly matches the generative model, and sufficient data are available for estimation.

The RNN produces an almost perfect match to the ground-truth model (right panel, orange line), with the KL divergence (per trial) between the true and predicted choice probabilities near zero (see Appendix A.6 for the definition of KL divergence). This close match is also theoretically expected: when the ground truth is an FQ-learning model with no individual differences, even the simplest RNN with linear units can exactly reproduce the same input–output behavior (see Appendix C). Therefore, it is unsurprising that an RNN, which uses hyperbolic tangent activation functions and can approximate linear responses in certain regimes, can learn behavior that is effectively equivalent to that of the FQ model.

Next, we consider a simple case involving individual differences. Figure 1B depicts a scenario in which the ground-truth agents follow the FQ-learning model, but with two distinct learning rates: half of the agents (Subjects 1 to 50) have a low learning rate ($\alpha = 0.1$), and the other half (Subjects 51 to 100) have a high learning rate ($\alpha = 0.9$). This setup instantiates the kind of situation discussed conceptually by Dezfouli et al. (2019). The left panels show data from a representative low learning rate agent, while the right panels show data from a high learning rate agent. As expected, the choice probabilities of the low learning rate agent change gradually, reflecting slower learning (left panel, gray line). In contrast, the high learning rate agent shows rapid fluctuations in choice probabilities, driven by recent outcomes (right panel, gray line).

The upper panels of Fig. 1B show predictions from the FQ-learning model fitted with a single, common parameter set (including learning rate) across all subjects (blue lines). Because this model must compromise between the two extreme learning rates among subjects, it adopts an intermediate learning rate. As a result, its choice probability (blue line) changes more rapidly than that of the low learning rate agents and more gradually than that of the high learning rate agents, leading to deviations from the true values in both cases. The KL divergences are approximately 0.05.

**Fig. 2:**
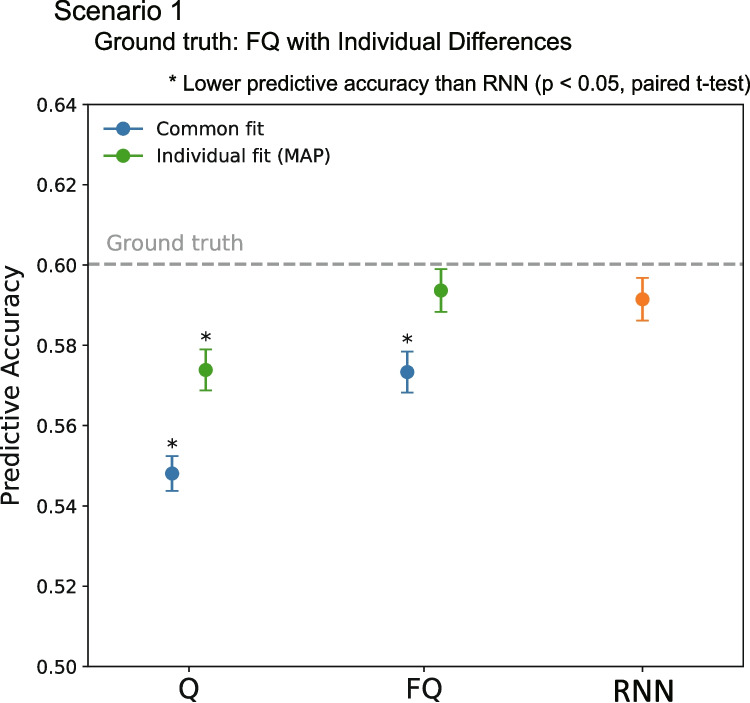
Comparison of the predictive accuracy of RL models and RNNs in a scenario where the true model is the FQ-learning model, with individual differences in learning rates (Scenario 1). In this scenario, half of the subjects have a learning rate of $\alpha = 0.1$, and the other half have a learning rate of $\alpha = 0.9$. The inverse temperature $\beta$ is fixed at 3.0 for all the subjects. Predictive accuracy is shown in terms of normalized likelihood for the actually chosen options. “*Q*” refers to standard *Q*-learning model, “FQ” to forgetting *Q*-learning model, and ‘RNN’ to a recurrent neural network with gated recurrent units (GRUs). The gray horizontal line represents the predictive accuracy based on the choice probabilities of the true model (FQ-learning). Error bars indicate standard errors of the mean. Asterisks ($*$) indicate models that perform significantly worse than the RNN (paired *t*-test, $p <.05$)

In the RNN model (bottom panels), there is an initial divergence between the true model and the RNN’s prediction (orange line) for the first 20 trials. However, beyond this point, the RNN effectively captures the overall trend of the true choice probability. This behavior demonstrates that the RNN effectively captures individual differences by leveraging information from earlier trials, storing this information in the latent units (see Supplementary Text S2, Figure S7, where we plot the latent variables). This mechanism can be regarded as the IDT property. Owing to this property, the KL divergence is less than 0.01.

While Fig. 1 shows the behavior of a single subject from each group, Supplementary Fig. S2 shows trial-by-trial choice probabilities for all subjects under conditions with individual differences, including both individual trajectories and group averages. The predicted choice probabilities of the RNN closely match the true model’s probabilities on average, which demonstrates that the RNN successfully captures individual differences and tracks changes in choice probability across subjects.

In the settings used in Fig. 1B, we consider a case with a large difference in learning rates (i.e., $\alpha = 0.1$ vs. 0.9). To explore how much individual difference is required for the RNN to begin adapting through IDT, we further examine this question in Supplementary Text S1, where we systematically vary the values of $\alpha$ of the ground-truth model (Fig. S5). We find that when the difference in learning rates reach approximately 0.4 (e.g., $\alpha = 0.3$ vs. 0.7), the predictive accuracy of the RNN improves as a result of IDT.

In addition to individual differences in the learning rate $\alpha$ (as shown in Fig. 1), we confirm that the RNN can also adapt to differences in the inverse temperature $\beta$ through the IDT mechanism (Supplementary Fig. S3).

## Impact of IDT on the Use of RNNs as Benchmarks for Cognitive Models: Illustrative Scenarios

We investigate how the IDT property of RNNs may influence conclusions about whether a fitted cognitive model (here, an RL model) sufficiently accounts for variability in choice behavior when RNNs are used as a benchmark. To this end, we present illustrative scenarios based on data generated through simulations, in which RL models are evaluated according to their predictive accuracy relative to an RNN and examine how the presence of IDT can affect the interpretation of such comparisons.

###  Scenario 1: When the FQ-learning Model is the True Model

In modeling an RL process in the two-armed bandit task, the standard *Q*-learning model, where the value of the unchosen option is not updated (i.e., $\alpha _F = 0$ in Eq. 2), is more commonly used than the FQ-learning model. On the other hand, several studies have reported that incorporating forgetting, as in the FQ model, can often improve model fit (Ito & Doya, 2009; Gershman et al., 2017; Katahira et al., 2017; Toyama et al., 2019b; Groman et al., 2019). Suppose a researcher first applies the standard *Q*-learning model to data. A key question is then whether the predictive accuracy based on test data can be regarded as sufficient, or whether there remains room for improvement. To answer this question, we consider whether an RNN can serve as a benchmark for evaluating the adequacy of a cognitive model.

We first consider a case the same as that shown in Fig. 1B, where the true underlying model is the FQ-learning model, and agents exhibit individual differences in learning rate: half of the subjects have a learning rate of 0.1, and the other half have a learning rate of 0.9. Each simulated subject completes two sessions; one session is used for training/fitting the RNN and *Q*-learning models, and the other session is used for testing the predictive performance. Predictive accuracy is quantified using the normalized likelihood, defined as the likelihood per trial on the test data (see Appendix A.6 for details).

Figure 2 shows the results. Let us focus first on the standard *Q*-learning model (denoted “*Q*”) shown on the left. The blue markers indicate the performance of the common fit, where a single parameter set is estimated for the entire group. This model yields significantly lower predictive accuracy than the RNN does (paired *t*-test, $p<0.05$; asterisks in the figure indicate models significantly worse than RNN). Even when using the individual fit (maximum a posteriori, MAP, see Methods for details), the standard *Q*-learning model fails to achieve accuracy comparable to that of the RNN ($p<0.05$). These results suggest that the standard *Q*-learning model lacks critical components necessary to capture the cognitive processes underlying behavior.

Next, suppose the researcher adds the FQ-learning model as a candidate model for fitting. In this case, the fitted model is identical in structure to the true generative model. Despite this, the common-fit FQ model yields significantly lower predictive accuracy than the RNN (Fig. 2, “FQ,” blue marker). This discrepancy can be attributed to the IDT property of RNNs: whereas the common-fit FQ model cannot account for individual differences, the RNN can implicitly capture such differences, thereby achieving higher predictive performance. Consequently, a researcher relying solely on the common fit might incorrectly conclude that the FQ model is inadequate.

In contrast, when the FQ model is fitted individually (using MAP estimation), its predictive performance matches or exceeds that of the RNN (green marker). In this case, comparing the individually fitted model to the RNN appears to be a reasonable approach. However, this outcome relies on the assumption that the IDT property allows the RNN to adequately capture individual differences. In the present example, this assumption may hold to some extent (except for the early trials): the RNN’s predicted choice probabilities closely match those of the ground truth (see Fig. 1B). Nonetheless, this assumption does not always hold. We examine such a case in the next scenario.

**Fig. 3:**
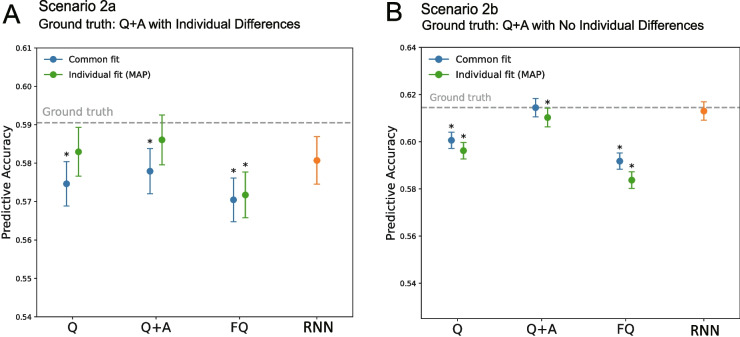
Comparison of RL models and RNNs in a scenario where the true model is an asymmetric learning rate model (*Q*+*A*), in which learning rates differ depending on the sign of the prediction error. **A** A case with individual differences where all the parameters are continuously distributed across the subjects (Scenario 2a). **B** A case without any individual differences, where all the parameters are fixed and shared across all the subjects (Scenario 2b). In Scenario 2a, the learning rates for positive and negative prediction errors ($\alpha ^+$ and $\alpha ^-$) are independently sampled from uniform distributions: $\alpha ^+ \sim \textrm{Uniform}(0.4, 0.9)$ and $\alpha ^- \sim \textrm{Uniform}(0.1, 0.6)$. The inverse temperature $\beta$ is sampled from $\textrm{Uniform}(1.0, 4.0)$. In Scenario 2b, the learning rates are fixed at $\alpha ^+ = 0.8$ and $\alpha ^- = 0.2$ for all the subjects, and the inverse temperature $\beta$ is fixed at 3.0. Error bars indicate the standard errors of the mean

### Scenario 2: When IDT Fails to Fully Capture Individual Differences

The previous scenario, in which the learning rates are one of two fixed values, represents an extreme case in terms of individual differences. In practice, it is more natural to assume that parameters such as the learning rate are continuously distributed across individuals within a population. The same applies to the inverse temperature parameter $\beta$, which governs the randomness of choice behavior. Moreover, when the true underlying model is not the FQ-learning model, the influence of past experiences on current choices can involve statistical interactions (Katahira, 2015), resulting in more complex dependencies between reward and choice histories. In such cases, the IDT property of RNNs is expected to yield an even more incomplete approximation of individual differences. Here, we examine a scenario in which the true model is the asymmetric learning rate model (simply referred to as the “*Q*+*A* model”), where the learning rate varies depending on the sign of the reward prediction error such that the *Q*-value of the chosen option $a_t$ is updated as follows:

4 $$\begin{aligned} Q_{t+1}(a_t)&= \left\{ \begin{array}{l} Q_t(a_t) + \alpha ^+ (r_t - Q_t(a_t)) \ \ \text {if} \ r_t - Q_t(a_t) \ge 0 \\ Q_t(a_t) + \alpha ^- (r_t - Q_t(a_t)) \ \ \text {if} \ r_t - Q_t(a_t) < 0 \\ \end{array} , \right. \end{aligned}$$

This model has been widely used in numerous studies that model human and animal learning behavior (Niv et al., 2012; Frank et al., 2007; Lefebvre et al., 2017; Palminteri et al., 2017). In this model, interactions between reward outcomes across trials can arise; for example, the influence of a reward received two trials ago may depend on whether a reward was received in the previous trial (Katahira, 2018). In this scenario, we do not assume any forgetting effect (i.e., $\alpha _F = 0$).

Figure 3A shows the predictive accuracy of RL models and the RNN when the true model is the *Q*+*A* model with individual differences (Scenario 2a). As expected, the highest predictive performance is achieved when the fitted model is the same as the true model (*Q*+*A*) and is fitted individually (Fig. 3A). The RNN achieves higher predictive accuracy than the common-fit *Q*+*A* model, presumably due to the IDT property. However, this improvement is modest and does not reach the level of the individual-fit *Q*+*A* model. Notably, when the standard *Q*-learning model is fitted individually, its predictive accuracy is not significantly different from that of the RNN ($t(99) = 1.614$, $p = 0.110$). This finding implies that if researchers focus on the predictive performance of individually fit models and use the RNN as a benchmark, they may mistakenly conclude that the standard Q-learning model is sufficient without ever considering the more appropriate Q+A model.

As shown above, the IDT property of RNNs does not always support the full capture of individual differences. While the significantly lower predictive accuracy of an individually fitted model compared to that of the RNN suggests that essential components may be missing from the model, it is important to emphasize that comparable or superior predictive performance of the individually fitted model relative to that of the RNN does not necessarily imply that the cognitive model is sufficient and leaves no room for improvement. Furthermore, the results from both Fig. 2 and Fig. 3 demonstrate that even when a common-fit cognitive model performs worse than the RNN does in terms of predictive accuracy, this discrepancy may be due to the RNN’s ability to exploit individual differences via the IDT property. Thus, inferior performance of the common-fit model does not necessarily indicate structural inadequacy of the cognitive model.

Figure 3B shows the results for a scenario in which the true model is the *Q*+*A* model, but no individual differences are assumed, that is, all agents share the same (ground truth) parameter values (Scenario 2b). In this case, the common-fit *Q*+*A* model achieves predictive accuracy comparable to that of the RNN. In contrast, the individual-fit *Q*+*A* model exhibits lower predictive performance compared to that of the common parameter model, likely due to increased estimation error arising from fitting each subject separately; when individual variability is sufficiently small, a common-fit (fixed-effect) model can reduce estimation noise and outperform individually fitted models in terms of prediction accuracy (Katahira, 2016). Notably, the RNN also achieves predictive accuracy equivalent to that of the true model in this case. This finding suggests that when a cognitive model with common parameters achieves predictive accuracy comparable to that of the RNN, the model may be considered adequate. However, such a conclusion relies on the assumption that the RNN has appropriately captured the underlying generative process. In practical applications with empirical data, the true process is unknown, and verifying whether the RNN has sufficiently captured it is generally a difficult task.

**Fig. 4:**
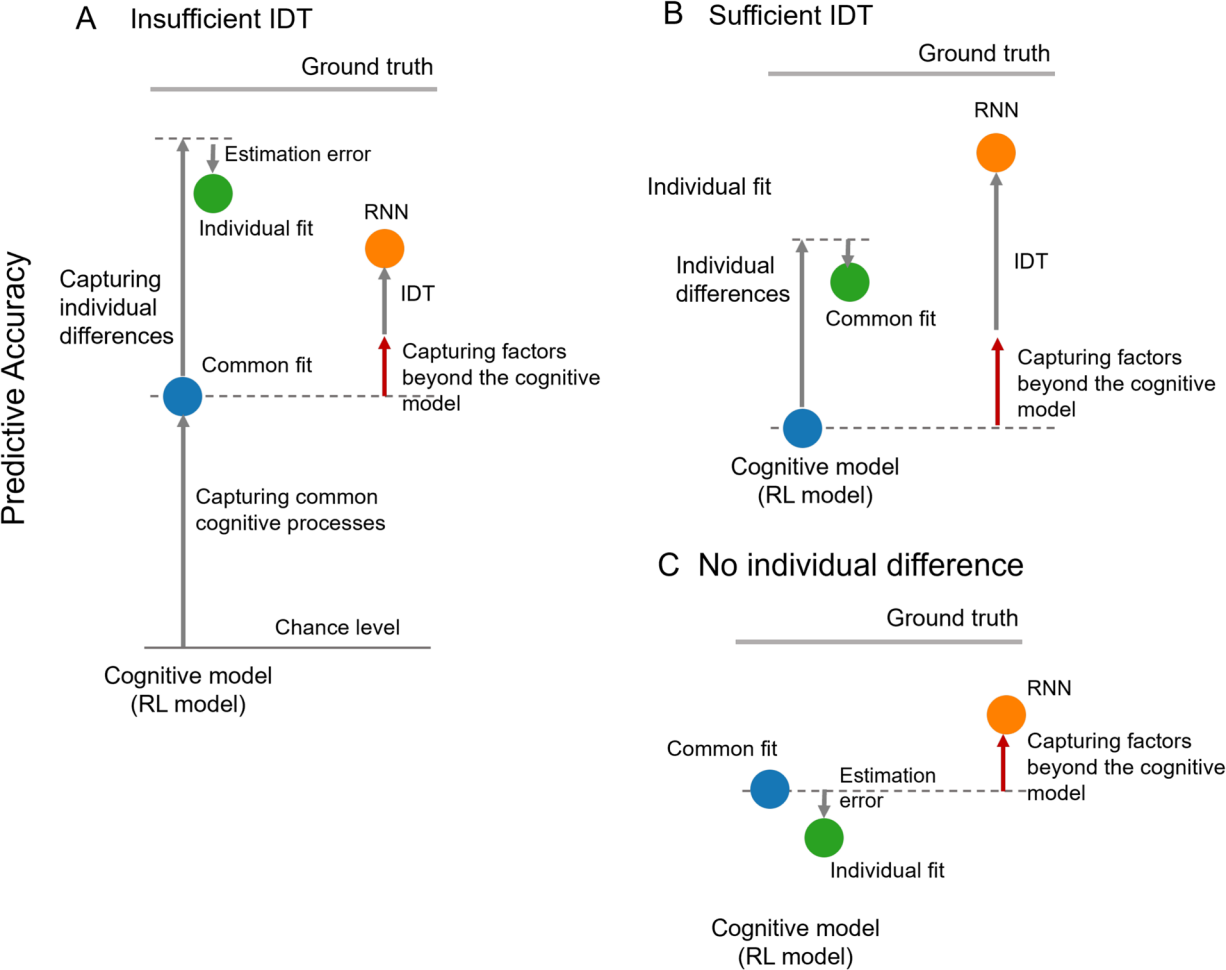
Schematic decomposition of predictive accuracy across different modeling approaches: a cognitive model with common parameters (common fit), a cognitive model with individual parameters (individual fit), and an RNN. **A** Case where the RNN performs better than the common-fit cognitive model but worse than the individual-fit model. **B** Case where the RNN achieves higher predictive accuracy than the individual-fit cognitive model does, assuming that IDT is sufficient. **C** Case where the common-fit cognitive model performs as well as or better than the individual-fit model does, suggesting that individual differences are negligible

### Decomposing Predictive Accuracy and the Influence of IDT

Based on the scenarios and simulation results described above, we summarize the factors that contribute to predictive accuracy and consider how the IDT property may influence the use of RNNs as benchmarks. The total variability in choice behavior can be separated into model-explainable and model-unexplainable components. The explainable component defines the upper bound of predictive accuracy achievable by the ground-truth model. The unexplainable component, which cannot be captured by any model, is irrelevant when comparing models. The key focus lies in how the explainable component is structured.

We decompose predictive accuracy, which is quantified as normalized likelihood on test data, into several contributing components (Fig. 4). Let us first consider the predictive accuracy of cognitive models (e.g., RL models; Fig. 4A). A common-fit cognitive model improves predictive accuracy by capturing cognitive processes that are shared across the population (“Capturing common cognitive processes”). An individually fitted model further enhances predictive accuracy by accounting for individual differences (“Capturing individual differences”). However, individually fitted models are more susceptible to estimation error, which can reduce predictive performance (“Estimation error”). Although common-fit models also suffer from estimation error, this effect is typically smaller due to the use of more data and is therefore neglected here.

Figure 4A illustrates a situation corresponding to the comparison between the Q+A model and the RNN in Scenario 2a, where the RNN outperforms the common-fit RL model but underperforms the individually fitted model. Two factors may account for the RNN’s higher predictive accuracy relative to that of the common-fit model. First, the RNN may better capture group-level cognitive processes that the common-fit model fails to represent (indicated by the red arrow), which is often the intended role of RNNs in cognitive modeling. Second, the RNN may improve prediction by leveraging its IDT property to capture individual differences (gray arrow).

However, it is difficult to distinguish these two factors. It is possible that the improvement results entirely from IDT-based adaptation to individual differences, without any advantage in capturing shared cognitive processes (i.e., the red arrow may be absent). Therefore, the fact that the RNN outperforms the common-fit model does not necessarily imply that the cognitive model lacks essential structural components.

Figure 4B corresponds to a case in which the RNN outperforms even the individual-fit model, as observed in Scenario 1 when evaluating the standard *Q*-learning model. In such cases, even if the RNN’s IDT accounts for individual differences to a similar extent as the individual-fit model, the remaining gain in predictive accuracy implies that the RNN is capturing aspects of the behavior that the cognitive model fails to represent. This suggests a structural limitation in the cognitive model.

Figure 4C corresponds to Scenario 2b, in which there are no individual differences in the data-generating process, as in the comparison with standard *Q*-learning or FQ-learning. In such cases, the predictive accuracy of the common-fit cognitive model is comparable to or even better than that of the individually fitted model, indicating that individual differences are negligible. Accordingly, IDT is unlikely to contribute to improved predictions by the RNN. Therefore, if the RNN still outperforms a cognitive model, it likely indicates that the cognitive model is missing key structural components.

## Assessing and Suppressing the IDT Property

### Evaluating IDT via On-Policy Simulation

The presence of IDT introduces uncertainty when interpreting the predictive accuracy of RNNs used as benchmarks for cognitive models. Therefore, it can be useful to assess whether an RNN can track individual differences (IDT), and if so, to what extent. For instance, if IDT can be sufficiently suppressed, it would justify a fair comparison between the RNN and common-fit cognitive models, as the RNN would more likely reflect only shared cognitive processes.

Here, we consider a method for assessing whether an RNN has acquired IDT. We adopt a heuristic approach based on an on-policy simulation (Dezfouli et al., 2019; also referred to as a closed-loop simulation), in which a trained RNN is used to generate new choice data by sampling actions according to its own predicted choice probabilities. The RNN receives input in the form of sequences of past choices and rewards (over 50 trials), generated from RL models with a plausible range of parameter values, in order to induce diversity in its latent states. We then fit an RL model to the simulated choice data and examine the distribution of the resulting parameter estimates. If the estimated parameters are narrowly concentrated around a single point despite the variability in input, this suggests that the RNN does not retain information about individual differences and therefore lacks IDT. For further details, see Appendix A.7.

**Fig. 5:**
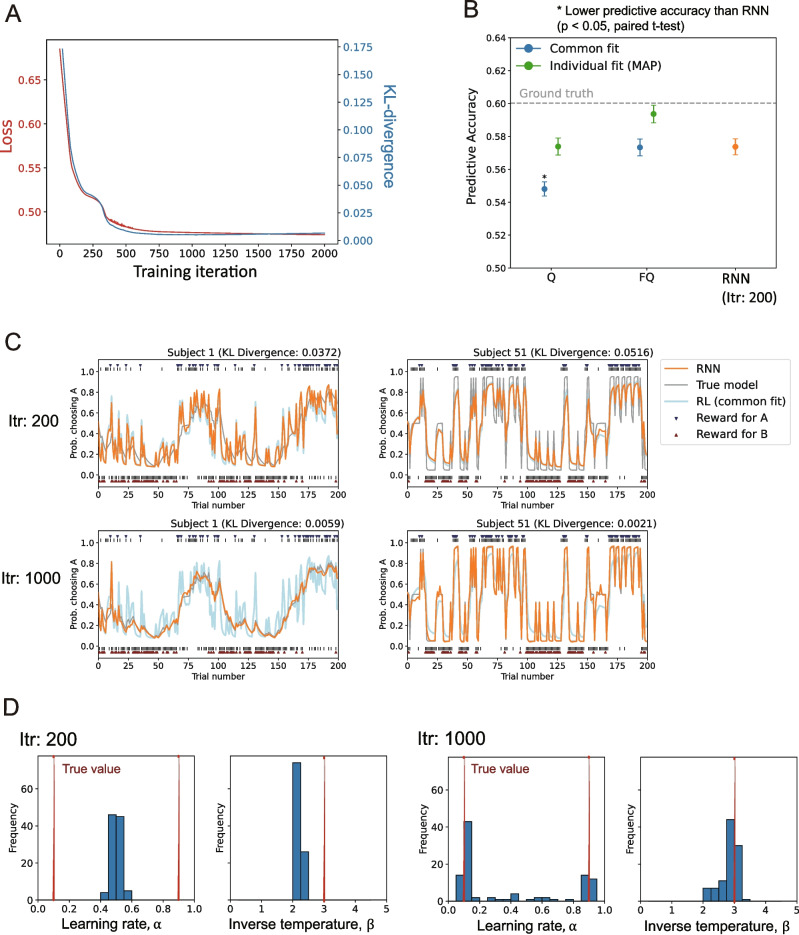
Relationship between training progression and IDT acquisition in Scenario 1. **A** Learning curves showing the RNN’s training progress, with the cross-entropy loss shown in red and the KL divergence (between the predicted and true choice probabilities) shown in blue. **B** Comparison between the RL models and the RNN trained up to training step 200. The convention is the same as that of Fig. 2. **C** Trajectories of the predicted choice probabilities at training steps 200 and 1000. Notably, at training step 200, the RNN’s predictions closely match those of the common-fit FQ model (shown in light blue). **D** Results of the on-policy IDT check for the RNNs at training steps 200 and 1000. The plots show the distribution of FQ-learning parameters fitted to simulated choice data generated by the RNN via on-policy simulation. The red vertical lines indicate the true parameter values used to generate the data ($\alpha = 0.1, 0.9$ and $\beta = 3.0$)

Figure 5D shows the results of the IDT check conducted on an RNN trained with data generated from the FQ-learning model, where individual differences exist only in the learning rate, $\alpha$, as in Scenario 1. After 1000 training iterations (right two panels in Fig. 5D), the KL divergence reaches its minimum, and the estimated values of $\alpha$ from the data generated by the RNN are distributed around the true values of 0.1 and 0.9, indicating that the RNN has stably maintained individual differences (Fig. 5D). This finding suggests that despite the RNN initially receiving input data generated from a broad range of learning parameters, its latent states ultimately converge, yielding a model that behaves stably according to one of the two distinct parameter values.

Figure 5A shows the learning curves of the loss function and KL divergence during training. Before converging to their asymptotic values, there is a phase in which learning temporarily plateaus, followed by a sharp decrease in loss. Figure 5C (top panels) shows the RNN’s predictions at 200 training iterations, corresponding to this plateau phase. Notably, at training step 200, the RNN’s predicted choice probabilities closely match those of the common-fit FQ-learning model (shown in light blue), suggesting that the RNN captures the group-level cognitive process during this stage.

In addition, during this stage, the on-policy IDT check reveals that the estimated learning rate parameters exhibit a unimodal distribution that is narrowly concentrated around 0.5 (Fig. 5D). This finding suggests that even after updating the latent state with 50 trials of off-policy simulation using agents with a wide range of parameter values, the effective learning rate in subsequent choices remains unchanged, providing evidence that the RNN has not yet acquired the IDT property.

These results indicate that, during training, the RNN first learns the common processes across all subjects, akin to a common-fit FQ-learning model, before later acquiring IDT as training progresses.

### Early Stopping of Training

As observed above, stopping RNN optimization at earlier training steps may suppress IDT. When using this RNN as a benchmark for model comparison, its predictive accuracy becomes comparable to that of the common-fit FQ model (Fig. 5B). This result suggests that, within the scope of common-fit models, the FQ-learning model may be considered a sufficient model.

However, early stopping may not only suppress IDT but also prevent the RNN from capturing common cognitive processes shared across the population. For example, consider the previously discussed scenario in which the true model is the *Q*+*A* model without individual differences (Scenario 2b). Supplementary Figure S3 shows the results of the on-policy IDT check for this scenario. The true learning rates were set to $\alpha ^+ = 0.8$ and $\alpha ^- = 0.2$. When the RNN was trained for 600 steps, the on-policy simulation confirmed that it successfully captured these parameters. However, at 100 training steps, the estimated $\alpha ^+$ and $\alpha ^-$ were narrowly concentrated around 0.3 to 0.4, indicating that the RNN failed to adequately represent the true underlying process.

In real-world applications, where the true generative model is unknown, it is difficult to determine whether an under-trained RNN, which does not account for individual differences, can still appropriately represent the common cognitive process of the population. One possible approach is to use the on-policy IDT check to identify the maximum number of training steps before IDT emerges. However, this method may be impractical, as each on-policy IDT check requires computationally intensive simulations and model fitting. Moreover, it remains unclear to what extent the pattern in which common cognitive processes are learned first, followed by individual differences, represents a general phenomenon. For these reasons, suppressing IDT through early stopping may not be a viable or practical approach when analyzing real-world data.

**Fig. 6:**
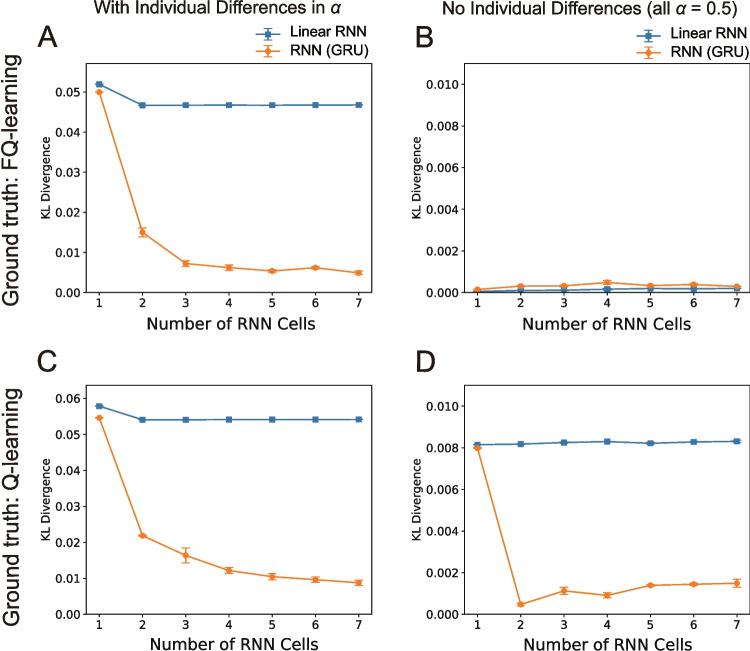
Relationship between the predictive accuracy and the number of RNN cells. The vertical axis represents the Kullback–Leibler (KL) divergence between the predicted choice probabilities of the RNN models (GRU and linear RNN) and the true probabilities; lower values indicate better predictive performance. The left panels correspond to scenarios with individual differences, while the right panels correspond to scenarios without individual differences. **A** Scenario in which the true model is the FQ-learning model with two learning rates groups: $\alpha = 0.1$ and $\alpha = 0.9$. IDT is considered to be acquired when the KL divergence falls below 0.05. **B** Scenario in which the true model is the FQ-learning model with no individual differences. **C** Scenario in which the true model is the standard *Q*-learning model with two learning rates groups: $\alpha = 0.1$ and $\alpha = 0.9$. **D** Scenario in the true model is the *Q*-learning model without individual differences. Error bars indicate the mean and standard error across five repetitions

### Effect of Reducing the Number of RNN Units on IDT Suppression

Another intuitive approach for suppressing IDT is to simplify the architecture of the RNN. One practical method involves reducing the number of hidden units, based on the rationale that representing individual differences as latent variables requires a sufficient number of units dedicated to capturing such variation.

The results of simulations examining this possibility are shown in Fig. 6. In addition to the GRU-based RNNs, we included results from linear RNNs, architectures in which the recurrent layer consists of purely linear units, as an example of simplified network structures. In panel A, the true model is an FQ-learning model with two distinct learning rates (as in Scenario 1). In this setup, a KL divergence of approximately 0.05 indicates that IDT has not been acquired, whereas values below this threshold suggest the presence of IDT (see Fig. 1). Contrary to expectations, even a small number of units (around 2 to 3) was already sufficient for the KL divergence to drop below the threshold, indicating the acquisition of IDT. Furthermore, in the absence of individual differences (panel B), we observe that when the true model is the FQ-learning model, even a single unit is sufficient to achieve near-zero KL divergence, as predicted by theory (Appendix C).

Panels C and D present results for the scenario in which the ground-truth model is the standard *Q*-learning model, where the values of unchosen options do not decay. In this scenario, interactions arise between past choices and rewards that are not easily captured by linear RNNs (Katahira, 2015). When there are individual differences in the learning rate $\alpha$, the pattern resembles that of the FQ-learning model (panel C). As shown in panel C, RNNs with two or more hidden units exhibit a marked decrease in KL divergence, indicating that IDT has been acquired. When there are no individual differences (panel D), models with two or more units also show a substantially lower KL divergence than those with a single unit. This contrast does not emerge in linear RNNs, suggesting that the nonlinearity of the RNN enables it to capture the history-dependent characteristics of *Q*-learning.

These findings indicate that IDT can emerge with as few as two units, and that multiple nonlinear units are necessary to adequately capture common cognitive processes. Thus, simply reducing the number of RNN units is unlikely to suppress IDT without compromising the flexibility that RNNs are expected to provide for modeling complex reinforcement learning processes.

## Empirical Demonstration Based on Real-World Datasets

We now present empirical examples of using RNNs as benchmarks for cognitive models, using real-world choice behavior data from a two-armed bandit task. The aim is to demonstrate how IDT should be accounted for when using RNNs for predictive benchmarking, rather than to replicate or reinterpret the original analyses. Accordingly, some aspects of our modeling and analysis procedures differ from those in the original studies. For instance, while the original studies did not employ cross-validation, we incorporate it here to evaluate the RNN’s predictive performance—none of the original studies used RNNs in their analyses.

The primary datasets analyzed here consist of human choice data from two-armed bandit tasks: the dataset from Sugawara and Katahira (2021) (referred to as the “Sugawara dataset,” $n = 143$), the dataset from Palminteri et al. (2017) (referred to as the “Palminteri dataset,” $n = 20$), and the dataset from Waltmann et al. (2022) (referred to as the “Waltmann dataset,” $n = 40$).

In these studies, the same participants engaged with multiple independent stimulus pairs (contexts), allowing us to split the data into training and test sets at the session (context) level. Additionally, a key contrast between the three datasets (Waltmann dataset vs. the others) is the substantial difference in the number of trials per context. For the details of these datasets, see Appendix A.8.

**Fig. 7:**
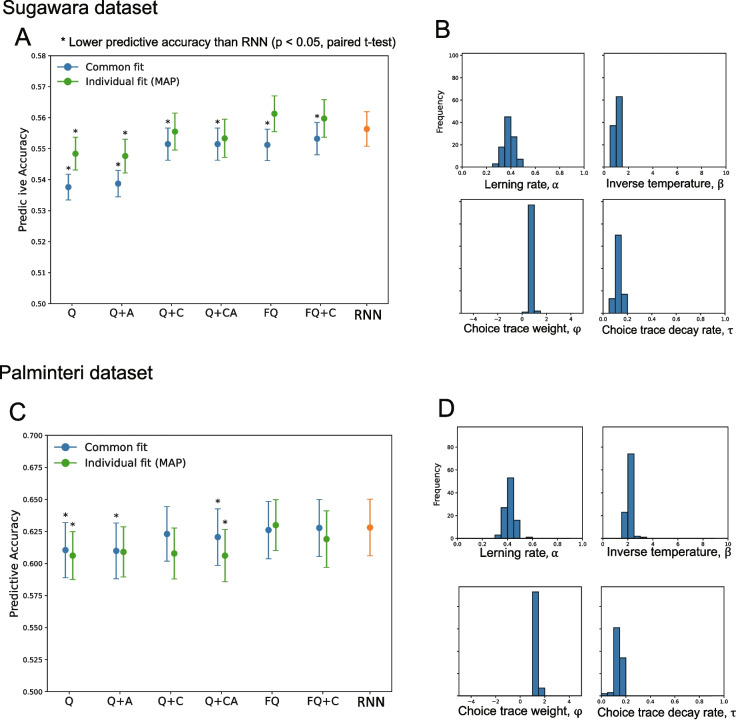
Empirical demonstration using real-world data. **A** and **B** Sugawara dataset; **C** and **D** Palminteri dataset. **A** and **C** Predictive accuracy (normalized likelihood) on the test data for various RL models and the RNN model. Each bar represents the mean predictive accuracy across participants. The error bars indicate the standard error of the mean (SEM). Asterisks indicate that the model performed significantly worse than the RNN did (paired *t* test, $p <.05$). **B** and **D** Results of the on-policy IDT check: Distribution of parameters estimated by fitting the FQ+C model to simulated choice data generated by the RNNs. The narrow distribution in all the panels suggests that the RNNs did not strongly acquire IDT in either dataset

### Sugawara and Palminteri Datasets

The experimental design of Sugawara and Katahira (2021) follows that of Palminteri et al. (2017), with essentially identical key features such as the number of trials and reward probabilities. In these experiments, trials from eight contexts are presented in an intermixed manner, with four contexts appearing in the first session and the remaining four in the second session. Each context consists of only 24 choice trials. This limited trial number per context may reduce the likelihood of RNNs developing strong IDT properties.

The predictive accuracies on the test data for various RL model variants and the RNN model for Sugawara dataset are shown in Fig. 7A. The *Q* and *Q*+*A* models with individually or common fit performed significantly worse than the RNN did. This result indicates that these two models are insufficient in terms of predictive accuracy and suggests room for improvement in their model structure. The *Q*+*C* and *Q*+CA models extend the *Q* and *Q*+*A* models, respectively, by incorporating choice hysteresis, accounting for the gradual influence of past choices (see Appendix A.2). These models were previously examined by Sugawara and Katahira (2021), and they demonstrated improved predictive accuracy compared to that of models without choice hysteresis (Q and Q+A models). We also evaluated the FQ-learning and FQ+C models, the latter of which incorporates both forgetting and choice hysteresis. The predictive accuracy for the individually fitted Q+C, Q+CA, FQ, and FQ+C models were comparable to or exceeded that of the RNN. However, under common fit, all of them exhibited significantly worse accuracy than the RNN.

To assess the degree to which the RNN acquired IDT, we performed on-policy IDT check using the RNN trained based on the Sugawara dataset. Specifically, we fitted the FQ+C model, which achieved the best predictive accuracy among the common-fit models, to data generated from the RNN. Figure 7B shows the distribution of parameter estimates of the fitted FQ+C model. The distribution was not particularly broad and concentrated on a single point, suggesting that the RNN did not strongly acquire IDT.

**Fig. 8:**
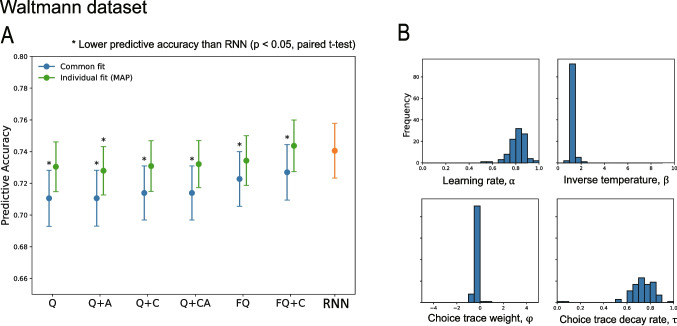
Empirical demonstration using real-world data (Waltmann dataset). **A** Predictive accuracy (normalized likelihood based on the test data) for various reinforcement learning models and the RNN model. The conventions are consistent with those in Fig. 7. **B** Results of the on-policy IDT check: Distribution of parameters estimated by fitting the FQ+C model to simulated choice data generated by the RNN. Compared with those in Fig. 7, the distributions of the learning rate $\alpha$ and choice trace decay parameter $\tau$ were slightly broader, suggesting the possibility that the RNN acquired a modest degree of IDT

In summary, while it is clear that standard Q-learning model without forgetting and the Q-learning model with asymmetric learning alone are insufficient, the common-fit RL models underperform the RNNs, which we assumed exhibited minimal IDT, suggesting that some cognitive component may be absent in the RL models we considered.

Figure 7C and D show the results for the Palminteri dataset. In contrast to the Sugawara dataset, the predictive accuracies of individually fitted models were lower than those of the corresponding common-fit models in most cases (4 out of 6 models). This finding suggests that individual differences were smaller in this dataset. As a result, the on-policy IDT check (Fig. 7D) also showed narrow parameter distributions, similar to those observed in the Sugawara dataset, indicating that the RNN likely did not acquire substantial IDT.

Considering that the predictive accuracy of the common-fit Q+C, FQ, and FQ+C models was not significantly lower than that of the RNN, there is no clear evidence that these RL models lack essential cognitive processes captured by the RNN. This result suggests that the FQ and FQ+C models may be sufficient for explaining choice behavior in this dataset. However, it is important to note that the Palminteri dataset included only 20 participants (compared to the 143 participants in the Sugawara dataset), and the lack of significant differences may simply reflect insufficient statistical power.

Overall, the normalized likelihoods were lower in the Sugawara dataset (0.54–0.56) compared to those in the Palminteri dataset (0.60–0.63). The Sugawara data were collected online, which may have resulted in some participants being inattentive or unengaged, leading to noisier and less predictable behavior (cf. Zorowitz et al., 2023). In contrast, the Palminteri data were collected in a laboratory setting, likely leading to greater engagement and more consistent behavior that is easier to model.

Regarding individual variability, participants in the Pal-minteri dataset had a relatively narrow age range (mean ± SD = 23.9 ± 0.7 years), whereas the Sugawara dataset included participants with a wider age range (38.7 ± 9.6 years), suggesting greater heterogeneity in the latter. On the other hand, both datasets involved only 24 trials per stimulus pair, which may have been insufficient for the RNN to acquire strong IDT properties.

Notably, the FQ and FQ-C models were not considered in the original studies (Palminteri et al., 2017; Sugawara & Katahira, 2021), and their performance, which was comparable to or even better than that of models with choice hysteresis, was unexpected. One possible explanation is that forgetting, by reducing the value of unchosen options, increases the tendency to repeat the same choice, thereby inducing the effective choice hysteresis.

### Waltmann Dataset

In the experiment by Waltmann et al. (2022), the primary aim was to assess the test–retest reliability of RL parameter estimates. Participants completed two sessions of a two-armed bandit task with the same underlying reward probability structure (though the visual stimuli representing the options differed), approximately one week apart. Each session consisted of 160 trials. We treated the first session as training data and the second session as test data (see Appendix A.8 for details).

Figure 8A shows the predictive accuracy of each model. The differences between RL models were relatively small compared to those in the Sugawara dataset, particularly between the Q and Q+A models and their counterparts incorporating choice hysteresis (Q+C and Q+CA; see Fig. 7 for comparison). When using the RNN as a benchmark, the overall pattern was consistent with that observed in the Sugawara data: while the Q, Q+C, Q+CA, FQ, and FQ+C models showed comparable predictive accuracy to that of the RNN under individual-fit approach, they performed significantly worse under common-fit approach.

The results of the on-policy IDT check (Fig. 8B) showed slightly broader distributions of the learning rate and choice trace decay parameter compared to those of the RNN trained based on the Sugawara and Palminteri datasets, suggesting that the RNN may have acquired a weak degree of the IDT property. Among the common-fit RL models, the FQ+C model had the highest predictive accuracy, but it still underperformed relative to the RNN. However, the RNN’s advantage may reflect its IDT property, leaving insufficient evidence to conclude that the FQ+C model is inadequate. Thus, it is possible that the FQ+C model is sufficient.

## Discussion

In this study, we demonstrated that RNNs, which are increasingly used to model behavior in both humans and animals, can capture individual differences in learning and decision-making processes. We refer to this property as individual difference tracking (IDT). This property arises from the RNN’s capacity for in-context adaptation, where past experiences are stored in latent states and influence subsequent predictions.

RNNs have attracted attention in computational cognitive modeling as models capable of representing more flexible processes than theory-based cognitive models can. However, the computations of RNNs remain largely opaque, making them fundamentally uninterpretable. In this regard, RNNs do not serve as replacements for cognitive models, which explicitly describe cognitive processes. In computational cognitive modeling, RNNs are considered to serve two primary roles. The first is to identify behavioral patterns not captured by the hypothesized cognitive models through simulations of an RNN trained based on empirical data (e.g., detecting oscillatory patterns in choice behavior; Dezfouli et al., 2019). Such insights can contribute to the formulation of hypotheses about necessary components in cognitive models. The second role is to provide a benchmark for evaluating whether a candidate cognitive model is sufficient or whether further refinement is necessary, effectively determining how much refinement a model requires.

Although the impact of IDT on the first role—discovering behavioral patterns through simulation—was beyond the scope of this study, our results on on-policy IDT check confirmed that individual differences could be partially reproduced in such on-policy simulations. This finding suggests that IDT may also influence such behavioral insights. In this paper, we mainly focused on the second role of RNNs and argued that the IDT property of such RNN models might provide an unfair benchmark when assessing predictive accuracy against that of theory-based cognitive models. In the following section, we discuss the implications of our findings, the influence of IDT in this context, and how researchers should consider using RNNs in cognitive modeling moving forward.

### Challenges in Comparing RNNs and Cognitive Models in Terms of Predictive Accuracy

RNNs are typically trained with a single set of parameters for an entire population. Similarly, theory-based cognitive models are often fit using a single parameter set pooled across all subjects (Dezfouli et al., 2019; Fintz et al., 2022; Eckstein et al., 2024). This common approach is particularly convenient in cross-validation settings, where the individuals in the training and test (or validation) sets differ, requiring reliance on population-level summary statistics (Dezfouli et al., 2019; Eckstein et al., 2024). In cognitive models, when parameters are shared across the population, the model is essentially incapable of adapting to individual differences—except in special cases, which we discuss later. This lack of adaptability gives RNNs a comparative advantage: When an RNN is compared with a cognitive model fit with common parameters, the RNN may appear to perform better—not because it captures cognitive processes more accurately but because it is able to adapt to individual differences through its IDT property. Consequently, even if the cognitive model has the correct structural assumption, it may seem inadequate, giving the false impression that it lacks essential components and requires further refinement.

Given that RNNs can express individual differences through IDT, one natural approach might be to compare them against cognitive models that also account for individual variability. This could be done by fitting models to individuals separately or by using hierarchical models where individual parameters are treated as random effects (Ahn et al., 2011; Daw, 2011). However, as we have observed in simulations with synthetic data, IDT does not perfectly capture individual differences. Consequently, simpler cognitive models fitted at the individual level may sometimes match or even exceed the predictive accuracy of RNNs, potentially leading researchers to overlook the need for further improvements in cognitive models (cf. Figure 3A).

### Interpreting RNN Benchmarks Under Uncertain IDT Property

Given the uncertainty about the extent to which an RNN exhibits IDT, the conclusions that can be drawn from using RNNs as benchmarks are summarized in the following two points: (1) If an individually fitted cognitive model underperforms relative to an RNN, it suggests that important components may be missing from the model (Fig. 4B). For example, in Scenario 2a (Fig. 3A; when the Q-learning model with asymmetric update was the ground truth), the individual-fit FQ-learning model underperformed compared to the RNN, indicating that it lacked the necessary cognitive component. However, the converse is not true: if an individually fitted cognitive model achieves predictive accuracy comparable to that of the RNN, this does not necessarily mean that the model is structurally sufficient. For example, in Fig. 3A, the standard Q-learning model outperforms the RNN when fitted individually, but this does not imply that the model is fully adequate. This outcome could arise because the RNN’s IDT is insufficient, preventing it from fully capturing individual differences. (2) If a common-fit cognitive model achieves predictive accuracy comparable to that of the RNN (Fig. 4C), it suggests that there may not be a significantly better cognitive model in terms of predictive accuracy. This situation is expected when individual differences are negligible or when constraints prevent the RNN’s IDT from functioning (e.g., in cases such as Fig. 3B, where no individual differences exist, the common-fit Q+A model and RNN show similar predictive accuracies). However, if the RNN itself does not sufficiently capture the true underlying process, it cannot be assumed that the RNN represents the upper bound of predictive accuracy.

As such, while the use of RNNs as benchmarks provides a rough reference point, importantly, the conclusions drawn from such benchmarks are inherently limited.

### Measuring the IDT Property in RNNs

As discussed above, for a fair comparison between RNNs and cognitive models, it is important to assess the extent to which the IDT property enhances the predictive accuracy. In this study, we examined a method to check for the presence of IDT using on-policy simulation (cf. on-policy IDT check). Specifically, we generated choice data from the trained RNN, fitted a cognitive model to the simulated data, and evaluated the distributions of the estimated parameters.

However, this method has several limitations. It is effective only when the individual differences encoded via IDT are stably maintained during the simulation. If the effective parameters fluctuate substantially, the resulting parameter estimates may not be meaningful. Moreover, the stability of the estimated parameters can rely heavily on the choice of cognitive model used for fitting. Additionally, the computational cost is substantial. On a standard laptop CPU, a single IDT check for one RNN requires several hours of computation. Therefore, the development of a more efficient and lightweight method for assessing IDT remains an important direction for future research.

### Challenges in Suppressing IDT

Two strategies have been explored to suppress IDT, but neither has proven fully effective. One approach is to stop training early (e.g., Fig. 5). While this approach can sometimes prevent IDT from being acquired, the appropriate stopping point is often unclear. Stopping too early may result in an RNN that fails to learn even the basic common cognitive processes, potentially performing worse than a common-fit cognitive model does. In our simulations, the RNN tended to learn group-level cognitive processes first, followed by adaptations to individual differences. However, this order may not always hold and likely depends on the model and task, suggesting that early stopping must be evaluated on a case-by-case basis.

Another strategy is to reduce the number of RNN units. Although this approach was expected to suppress IDT, we found that even with only two units, IDT still emerged. Thus, reducing the number of units may not be an effective solution. Similarly, using a linear activation function made the acquisition of IDT less likely. In this setting, the RNN was able to replicate the input—output mapping of an FQ-learning model but failed to capture the behavior of the standard Q-learning model when it served as the ground truth. This finding suggests that linear RNNs lack the flexibility to model more complex behavioral patterns. In such cases, using an RNN provides no clear advantage.

Reducing the number of trials per session is another potential approach. Since IDT depends on past behavior, shorter sessions may make it more difficult for the RNN to acquire IDT. Evidence of this effect was observed using the Sugawara and Palminteri datasets. However, this strategy requires an experimental design with short sessions and multiple contexts, which is not always feasible. Once data collection is complete, it is difficult to apply this approach retrospectively through post hoc analyses.

Overall, it appears difficult to suppress IDT without compromising the RNN’s ability to capture common cognitive processes. Furthermore, cognitive models with common parameters do not always accurately capture representative behavioral patterns across individuals. For example, if participants exhibit opposing asymmetries in learning rates, averaging across individuals could falsely suggest symmetry, thereby masking meaningful individual-level structures.

### Future Directions: Expanding RNNs to Better Represent Individual Differences

As discussed above, suppressing IDT through a single method may not always be feasible. Rather than focusing on eliminating IDT, exploring ways to explicitly model individual differences within RNNs may be more productive. Neural network architectures that aim to explicitly model individual differences via RNNs have been proposed (Dezfouli et al., 2019; Song et al., 2021). For example, the encoder–decoder architecture developed by Dezfouli et al. (2019) involves an encoder that maps an individual’s behavioral data onto a low-dimensional latent space representing individual-specific characteristics. These latent variables are then passed through a decoder, which outputs the connection weights of an RNN. The resulting RNN models the individual’s behavior and can be used to predict future actions. The results obtained by Dezfouli et al. (2019), both in simulations using synthetic data and in applications to real behavioral data, were promising. However, as we demonstrated in the present study, RNNs may themselves acquire the ability to represent individual differences via IDT. This raises the possibility that individual variability could be encoded directly within the RNN itself, potentially bypassing the intended role of the latent variables. Whether such latent variables can reliably capture individual differences across a wide range of conditions remains an open question.

The development of RNN-based models that can explicitly and sufficiently represent individual differences, whether through latent variables or alternative mechanisms, is an important direction for future research.

### Tracking Within-Subject Parameter Variability

In this study, we interpreted the RNN’s ability to dynamically adjust effective parameters such as the learning rate within a session—through in-context learning—as a form of adaptation to individual differences. However, it is also plausible that parameters vary within individuals over time, and as demonstrated in Appendix D, RNNs are capable of tracking such changes. This ability may be more appropriately described as *individual state tracking* rather than IDT.

Ultimately, such within-subject variability may be better modeled explicitly within cognitive models. Indeed, numerous cognitive models have been proposed that allow parameters such as the learning rate or inverse temperature to change during task performance. Some of these models assume stochastic fluctuations introduced by noise (Samejima et al., 2005; Ito & Doya, 2009; Findling et al., 2019), whereas others define specific rules governing the temporal dynamics of parameter change (Yechiam et al., 2005; Bai et al., 2014; Piray & Daw, 2024).

When RNNs outperform cognitive models with stationary parameters, it may indicate that such within-subject parameter dynamics are relevant, and incorporating them into the cognitive model could lead to improved explanatory power.

### The Boundary Between IDT and Within-Subject Cognitive Processes

Throughout this paper, we have implicitly assumed that the components described in cognitive models (i.e., RL models) are intended to reflect cognitive processes within individuals, whereas in-context adaptation by RNNs reflects individual differences and is thus conceptually distinct from these cognitive processes. While this distinction is clear at the definitional level, the boundary between them is often uncertain in practice. Some mechanisms expressed in cognitive models may themselves be interpreted as forms of in-context adaptation to individual differences. For example, choice hysteresis in RL models (Eq. 7 in Appendix A.2) can be viewed as a form of in-context adaptation, in which a latent variable tracks past choices and influences future decisions. In some cases, choice hysteresis may genuinely reflect within-subject cognitive dynamics; in others, it may simply serve to capture stable individual differences.

Consider a situation in which an RL model assumes no initial bias in choices (i.e., identical initial *Q*-values for both options), but some participants exhibit strong initial preferences. In such cases, incorporating a choice hysteresis effect can improve the predictive accuracy by accounting for this bias. For example, if a participant consistently selects option B from the beginning, a model with choice hysteresis may predict continued selection of option B. When these initial biases differ across individuals, such in-context adaptations effectively capture individual differences—implying that the RL model itself has an IDT property.

Thus, whether a mechanism in a cognitive model constitutes IDT depends on both the structure of the model and the nature of the underlying process. Researchers must therefore be explicit about which components they regard as part of the cognitive process and how these components are implemented in the model.

Moreover, in the presence of model misspecification, mechanisms defined as cognitive components may, in effect, serve the role of IDT. Awareness of this possibility is crucial when interpreting modeling results. For example, in the situation described above, one could address the issue by including a free parameter for initial choice bias or allowing initial *Q*-values to vary across participants (e.g., Zhu et al., 2025).

## Conclusion

We have examined how the ability of a single RNN to adapt to individual differences affects its predictive accuracy as a benchmark for cognitive models. The impact of this IDT property is likely to vary depending on multiple factors, such as the design of the behavioral task, the magnitude of individual differences, and the assumed cognitive model structure, making it difficult to establish general guidelines. At present, researchers should be aware of this property when using RNNs for behavioral modeling. Further understanding of the IDT property and other characteristics of RNNs is needed, along with continued efforts to harness these properties to make RNNs a more useful tool in computational cognitive modeling.

## Supplementary Information

Below is the link to the electronic supplementary material.

Supplementary file 1 (pdf 1255 KB)

## Figures and Tables

**Fig. 9:**
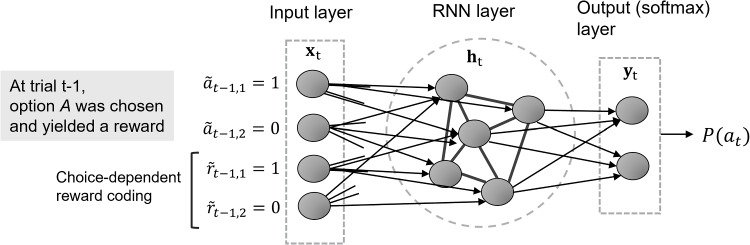
Schematic diagram of RNN models. The input consists of choice and reward information from the previous trial, with choice represented via one-hot encoding and reward coded via choice-dependent reward coding. These inputs are fed into an RNN layer with recurrent connections, where they are integrated with past history data before being passed to the output layer. In the output layer, the probability of selecting each option is computed via the softmax function

**Fig. 10:**
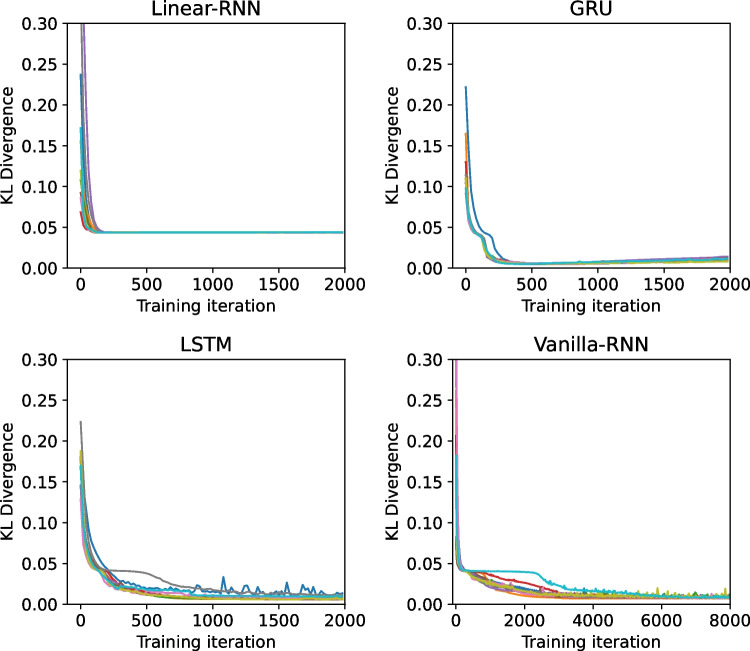
Comparison of learning curves across RNN architectures. The average KL divergence, which represents the deviation from the true model, is plotted. When the KL divergence drops below 0.05 and approaches zero, it is considered to have entered the IDT mode. The results starting from ten different initialized network weights are overlaid in different colors. The simulation settings are the same as those in Fig. 1B

**Fig. 11:**
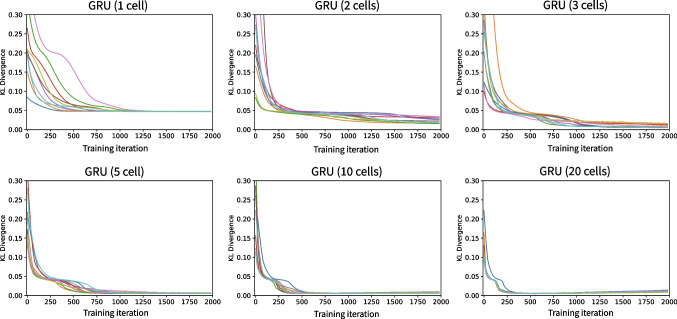
Comparison of the learning curves across different numbers of GRU units. The number of GRU units was varied as follows: 1, 2, 3, 5, 10, and 20. The KL divergences between the true model and RNN were plotted. When the KL divergence was below 0.05 and approached zero, the model was considered to have entered the IDT mode. The results from ten different random initializations of network weights are overlaid in different colors. The simulation settings were identical to those used in Fig. 1B

**Fig. 12:**
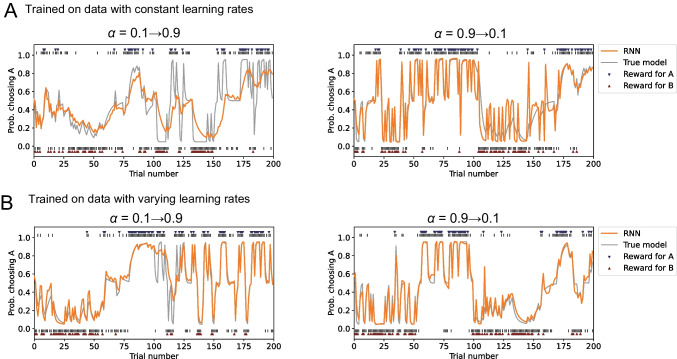
Tracking within-subject changes in the learning rate by the RNN. **A** A case where the RNN was trained based on data with a constant learning rate and tested using data with a switching learning rate. The RNN was trained based on simulated data with agents whose learning rate $\alpha$ remained constant throughout the task (either $\alpha =0.1$ or $\alpha =0.9$) and tested using data with agents whose learning rate switched at trial 101 (from 0.1 to 0.9 on the left, from 0.9 to 0.1 on the right). **B** A case with both training and test sets containing learning rate switches. The RNN was trained based on agents whose learning rate changed within the task in the same manner as the test data. The orange lines represent the RNN’s predicted choice probability, and the gray lines show the ground-truth choice probability generated by the FQ-learning model

## Data Availability

The datasets used in this study are publicly available from the following sources: The data from Sugawara and Katahira (2021) are available at https://figshare.com/articles/Cognitive_bias_and_perseverance/10042319. The data from Waltmann et al. (2022) are available at https://osf.io/4ng3e/. The data from Palminteri et al. (2017) are available at https://figshare.com/articles/dataset/Confirmation_bias_in_human_reinforcement_learning_evidence_from_counterfactual_feedback_processing/5220619?file=8916295

## Appendix A: Methods

### A.1 Task Settings for Simulations

We simulated the choice behavior of 100 agents (virtual subjects) on a two-armed bandit task via various RL models with parameters (e.g., learning rate) that were systematically varied.

In the two-armed bandit task used for the simulations, one option was associated with a high reward probability, 0.7, whereas the other option was associated with a low reward probability, 0.3. At each trial *t* (where *t* denotes the trial index), a reward was given ($r_t = 1$) based on the probability associated with the chosen option; otherwise, no reward was given ($r_t = 0$). After each 50-trial block, the reward probabilities of the two options were reversed. For each trial, whether a reward was available for each option was predetermined according to these probabilities. Although the same reward sequence was used across agents, different sequences were used for the training and test data. In all the simulations conducted in the present study, each agent completed two sessions of 200 trials, resulting in three reversals per session. One session was used as training data for the RNN and RL models, whereas the other session was used as test data to evaluate the predictive accuracy. All simulations were implemented in Python (version 3.12.1).

### A.2 Reinforcement Learning (RL) Models

Here, we first provide the general formulation of the variants of the Q-learning model considered in the present study. This model includes various additional components (in addition to the standard Q-learning model), such as an asymmetric learning rate, forgetting rate, and choice autocorrelation (choice hysteresis). Specific reduced models are derived from this general formulation by decreasing or fixing the parameters.

The *Q*-value or action value, $Q_t(a_t)$, for the chosen option $a_t \in \{A, B\}$ at trial *t* is updated as

5 $$\begin{aligned} Q_{t+1}(a_t)&= \left\{ \begin{array}{l} Q_t(a_t) + \alpha ^+ (r_t - Q_t(a_t)) \ \ \text {if} \ r_t - Q_t(a_t) \ge 0 \\ Q_t(a_t) + \alpha ^- (r_t - Q_t(a_t)) \ \ \text {if} \ r_t - Q_t(a_t) < 0 \\ \end{array} , \right. \end{aligned}$$

where $$\alpha ^+ \in [0,1]$$ and $$\alpha ^- \in [0,1]$$ are the learning rates that determine how the model updates the *Q*-value depending on the sign of the RPE, $$r_t - Q_t(a_t)$$. The initial *Q*-values are set to zero (i.e., $$ Q_1(A) = Q_1(B) = 0$$).

The *Q*-value for the unchosen option $\bar{a}_t \in \{A, B\}$ is assumed to decay as follows:

6 $$\begin{aligned} Q_{t+1}(\bar{a}_t) = (1 - \alpha _F) Q_t(\bar{a}_t), \end{aligned}$$

where $\alpha _F$ is the forgetting rate, which determines the rate at which the value of the unchosen option decays.

To model the effects of choice history (choice hysteresis), the choice trace (or choice kernel) $C_t(i)$, which determines how frequently option *i* has been chosen recently, is computed as follows (Wilson & Collins, 2019):

7 $$\begin{aligned} C_{t+1}(i) = (1-\tau ) C_t(i) + \tau I(a_t = i), \end{aligned}$$

where the indicator function $I(\cdot )$ is 1 if the statement is true and 0 if the statement is false. The initial values are set to zero, i.e., $C_1(A) = C_1(B) = 0$. The parameter $\tau \in [0,1]$ is the decay rate of the choice trace.

The choice probability (for option A) is determined by the softmax function:

8 $$\begin{aligned} P(a_t = A) = \frac{1}{1+\exp \left( - \beta ( Q_t(A) - Q_t(B) ) + \varphi (C_t(A) - C_t(B))\right) }. \end{aligned}$$

The parameter $\beta \in [0,\infty )$ is the inverse temperature, which indicates how sensitively the choice probability changes with the value difference between options. A larger $\beta$ results in a more sensitive change in the choice probability. The choice trace weight $\varphi \in (-\infty ,\infty )$ controls the tendency to repeat (when $\varphi > 0$) or avoid (when $\varphi < 0$) recently chosen options. Since the model involves only two options, the choice probability for option B is given by $P(a_t = B) = 1 - P(a_t = A)$.

First, we consider six variants of Q-learning models without forgetting ($\alpha _F = 0$). The standard Q-learning model (Q-model) assumes symmetric learning rates, $\alpha ^+ = \alpha ^- = \alpha$, and does not include a choice autocorrelation factor ($\varphi = 0$). The Q+C model extends the Q-model by incorporating a choice trace component, allowing $\varphi$ and $\tau$ to be estimated as free parameters while still assuming symmetric learning rates. The Q+A model introduces asymmetric learning rates, allowing $\alpha ^+$ and $\alpha ^-$ to differ, but does not include a choice trace component ($\varphi = 0$). The Q+CA model combines both features, allowing asymmetric learning rates and including a choice trace mechanism.

In addition, we consider forgetting versions of the Q-learning model, termed FQ and FQ+C. These models are equivalent to the Q and Q+C models, respectively, but the forgetting rate $\alpha _F$ is equal to the learning rate $\alpha$.

The parameters of the ground-truth RL models used to generate the data are provided in the captions of the corresponding figures.

### A.3 Fitting Reinforcement Learning Models

We fitted RL models to synthesized and empirical choice data using two approaches: common parameter fitting and individual parameter fitting (hereafter referred to as “common fit” and “individual fit,” respectively). In the common fit approach, we assumed that all the subjects share the same parameter values and thus estimated a single set of parameters for the entire dataset via maximum likelihood estimation, where the parameters were optimized by minimizing the total negative log-likelihood across all subjects. In contrast, in the individual fit approach, we allowed model parameters to vary across subjects and fitted them separately for each subject using maximum a posteriori (MAP) estimation. This method combines the log-likelihood of the data with prior distributions over the parameters, providing regularization and improving estimation stability, especially when the number of trials per subject is limited (Katahira, 2016).

To account for individual differences in parameters, one can either maximize the likelihood for each individual or use hierarchical models that estimate both individual-level parameters and group-level distributions (Daw, 2011). In the present study, we adopted MAP estimation (as in Palminteri et al. (2017); Sugawara and Katahira (2021)). While hierarchical modeling generally yields more accurate parameter estimates, it typically requires computationally intensive procedures such as Markov chain Monte Carlo (MCMC) sampling (Ahn et al., 2011) or the expectation–maximization (EM) algorithm (Huys et al., 2011). These procedures can be time-consuming, especially when performing systematic simulations. Moreover, hierarchical models can be sensitive to the prior distributions and model specifications. In some cases, strongly informative priors may lead to excessive shrinkage, resulting in unstable estimates and requiring additional effort for model tuning (Sumiya & Katahira, 2020). Maximum likelihood estimation, which does not rely on prior assumptions, tends to produce larger estimation errors and generally yields lower predictive accuracy than MAP estimation does. When weakly informative priors are used, MAP estimation can achieve predictive performance comparable to that of fully hierarchical Bayesian models (Katahira, 2016). Therefore, the results obtained using MAP estimation can be expected to approximate those of hierarchical models.

We used the same prior distribution as that used in Sugawara and Katahira (2021), which follows that in Palminteri et al. (2017). Specifically, the learning rate parameters were assigned $\textrm{Beta}(1.1, 1.1)$ priors (either symmetric $\alpha$ or asymmetric $\alpha ^+$ and $\alpha ^-$), the inverse temperature parameter $\beta$ was given a $\textrm{Gamma}(1.2,\ \text {scale}=5.0)$ prior, and the choice trace weight $\varphi$ was assigned a Gaussian prior $\mathcal {N}(0,\ \sigma ^2=5)$. The decay parameter of the choice trace $\tau$ was, if included, also assigned a $\textrm{Beta}(1.1, 1.1)$ distribution. If the initial *Q* values were treated as free parameters, uniform priors over [0, 1] were implicitly imposed via bounded optimization. For both approaches, we employed the sequential least squares programming (SLSQP) algorithm, implemented in the scipy.optimize.minimize function from the Python SciPy package, to perform constrained optimization with five random initializations to avoid local minima.

### A.4 RNN Architectures

The RNN architectures examined in this paper consist of an input layer that receives the action and reward at trial $t-1$, an output layer that generates the choice probability for trial *t*, and an RNN layer in between (Fig. 9). This structure is standard in previous studies that have used RNNs to model RL processes (e.g., Dezfouli et al., 2019), except for the method of coding the reward in the input layer, which will be described below.

#### Input Layer

The input to the network at trial *t* is represented by the four-dimensional vector

9 $$\begin{aligned} \textbf{x}_t = \begin{bmatrix} \tilde{a}_{t-1,1} \\ \tilde{a}_{t-1,2} \\ \tilde{r}_{t-1,1} \\ \tilde{r}_{t-1,2} \end{bmatrix}. \end{aligned}$$

Here, $\tilde{a}_{t-1}$ is a one-hot vector such that if option A is chosen, the first element, $\tilde{a}_{t-1,1}$, is 1, and the second element, $\tilde{a}_{t-1,2}$, is 0. If option B is chosen instead, $\tilde{a}_{t-1,1} = 0$ and $\tilde{a}_{t-1,2} = 1$. $\tilde{r}_{t-1}$ is a vector defined as follows: if the *i*-th option is chosen at trial $t-1$, the *i*-th element, $\tilde{r}_{t-1, i}$, has a reward value of $r_{t-1}$. The *j*-th element corresponding to the unselected *j*-th option is set to zero. This reward coding scheme is referred to as *choice-dependent reward (CDR) coding*.

Many existing studies input the reward value $r_t$ as a single scalar value, regardless of the choice. This is referred to as *choice-independent reward (CIR) coding*. In CIR coding, the network needs to learn the interaction between reward and choice, whereas in CDR coding, this is not necessarily needed, which makes training more efficient. Additionally, as demonstrated in Appendix C, CDR coding allows a linear RNN to be equivalent to an FQ-learning model.

#### RNN Layer

In this study, we primarily used a gated recurrent unit (GRU; Cho, 2014) as the recurrent layer of the RNN for the following reasons. GRUs offer a simpler architecture than long short-term memory (LSTM; Hochreiter, 1997) while achieving comparable performance. Unlike LSTMs, which maintain two latent states per unit, GRUs use only a single latent variable per unit. Although LSTMs were initially used to model reward-based choice behavior (Dezfouli et al., 2019), GRUs have become more commonly used in recent work (Ger et al., 2024a, b). Simpler architectures such as the vanilla RNN have also been applied to choice modeling, but they suffer from slow convergence (see Appendix B).

We also considered a further simplified variant, a linear RNN, as a point of comparison. The definitions of each recurrent unit type—vanilla RNN, linear RNN, LSTM, and GRU—are provided below, in that order.

Let $N_h$ denote the number of units in the RNN layer.

##### Vanilla RNN

In the vanilla RNN, the internal state vector $\textbf{h}_t$ (an $N_h$-dimensional vector) is updated, and the output is calculated as follows:

10 $$\begin{aligned} \textbf{h}_t&= \tanh (\textbf{W} \textbf{x}_t + \textbf{U} \textbf{h}_{t-1} + \textbf{b}_h). \end{aligned}$$

Here, the function $\tanh (\cdot )$ is the hyperbolic tangent activation function, which introduces nonlinearity to the model. The matrix $\textbf{W}$, which has dimensions $N_h \times 4$, is the weight matrix that is applied to the input $\textbf{x}_t$, while $\textbf{U}$ is the weight matrix, with dimensions $N_h \times N_h$, applied to the previous latent state $\textbf{h}_{t-1}$. The vector $\textbf{b}_h$, which is $N_h$-dimensional, is the bias associated with the latent state.

##### Linear RNN

The linear RNN is obtained by replacing the $\tanh$ function in Eq. (10) with the identity function as follows:

11 $$\begin{aligned} \textbf{h}_t = \textbf{W} \textbf{x}_t + \textbf{U} \textbf{h}_{t-1} + \textbf{b}_h. \end{aligned}$$

##### GRU

In GRU (Cho, 2014), the internal state vector $\textbf{h}_t$ is updated as follows:

12 $$\begin{aligned} \textbf{z}_t&= \sigma (\textbf{W}_z \textbf{x}_t + \textbf{U}_z \textbf{h}_{t-1} + \textbf{b}_z), \end{aligned}$$

13 $$\begin{aligned} \textbf{r}_t&= \sigma (\textbf{W}_r \textbf{x}_t + \textbf{U}_r \textbf{h}_{t-1} + \textbf{b}_r), \end{aligned}$$

14 $$\begin{aligned} \tilde{\textbf{h}}_t&= \tanh (\textbf{W}_h \textbf{x}_t + \textbf{U}_h (\textbf{r}_t \odot \textbf{h}_{t-1}) + \textbf{b}_h), \end{aligned}$$

15 $$\begin{aligned} \textbf{h}_t&= (1 - \textbf{z}_t) \odot \textbf{h}_{t-1} + \textbf{z}_t \odot \tilde{\textbf{h}}_t, \end{aligned}$$

where $\textbf{z}_t$ is the update gate, $\textbf{r}_t$ is the reset gate, $\tilde{\textbf{h}}_t$ is the candidate latent state, $\sigma$ is the sigmoid function, and $\odot$ represents elementwise multiplication.

##### LSTM

The LSTM (Hochreiter, 1997) update equations are as follows:

16 $$\begin{aligned} \textbf{f}_t&= \sigma (\textbf{W}_f \textbf{x}_t + \textbf{U}_f \textbf{h}_{t-1} + \textbf{b}_f), \end{aligned}$$

17 $$\begin{aligned} \textbf{i}_t&= \sigma (\textbf{W}_i \textbf{x}_t + \textbf{U}_i \textbf{h}_{t-1} + \textbf{b}_i), \end{aligned}$$

18 $$\begin{aligned} \textbf{o}_t&= \sigma (\textbf{W}_o \textbf{x}_t + \textbf{U}_o \textbf{h}_{t-1} + \textbf{b}_o), \end{aligned}$$

19 $$\begin{aligned} \tilde{\textbf{c}}_t&= \tanh (\textbf{W}_c \textbf{x}_t + \textbf{U}_c \textbf{h}_{t-1} + \textbf{b}_c), \end{aligned}$$

20 $$\begin{aligned} \textbf{c}_t&= \textbf{f}_t \odot \textbf{c}_{t-1} + \textbf{i}_t \odot \tilde{\textbf{c}}_t, \end{aligned}$$

21 $$\begin{aligned} \textbf{h}_t&= \textbf{o}_t \odot \tanh (\textbf{c}_t), \end{aligned}$$

where $\textbf{c}_{t-1}$ is the cell state from the previous time step, $\textbf{f}_t$ is the forget gate, $\textbf{i}_t$ is the input gate, $\textbf{o}_t$ is the output gate, and $\tilde{\textbf{c}}_t$ is the candidate cell state

In all the RNN models, the number of latent units was generally fixed at 10 because increasing the size beyond this point did not result in notable improvements in prediction accuracy (see Appendix B).

#### Output Layer

In the output layer, the state of the RNN layer, $\textbf{h}_t$, is linearly transformed as follows:

22 $$\begin{aligned} \textbf{y}_t&= \textbf{W}_{y} \textbf{h}_t + \textbf{b}_y. \end{aligned}$$

Here, $\textbf{y}_t$ is a 2-dimensional vector representing the output for the two possible choices, and $\textbf{b}_y$ is a 2-dimensional bias vector. The matrix $\textbf{W}_y$ is an $2 \times N_h$ matrix that linearly transforms the latent state $\textbf{h}_t$.

The choice probability of option A is then determined via the softmax function:

23 $$\begin{aligned} P(a_t = A)&= \frac{1}{1 + \exp \left( -\left[ y_{t,1} - y_{t, 2} \right] \right) }. \end{aligned}$$

Here, $y_{t, i}$ denotes the *i*-th element of the vector $\textbf{y}_t$.

#### Loss Function Used for Training RNNs

Categorical cross-entropy was employed as the loss function, which corresponds to maximum likelihood estimation. For a given target choice label $a_t^s$ (where *s* indexes the subject) and the predicted choice probabilities $P_{\text {pred}}(a_t^s)$ for options A and B, the loss is defined as:

24 $$\begin{aligned} \mathcal {L}_{\text {CE}}&= - \big [ I(a_t^s = A) \log \left( P_{\text {pred}}(a_t^s = A) \right) \nonumber \\&\quad + I(a_t^s = B) \log \left( P_{\text {pred}}(a_t^s = B) \right) \big ], \end{aligned}$$

where $I(\cdot )$ is the indicator function. All subjects’ data were used as a single batch during training.

### A.5 Implementation and Training of the RNN Models

The vanilla and linear RNNs were constructed using the SimpleRNN class from Keras implemented in TensorFlow (version 2.16.1). Similarly, the GRU and LSTM models were built using the GRU and LSTM classes.

To train the networks, the adaptive moment estimation (Adam) optimizer was used with a constant learning rate of 0.001. Additionally, gradient clipping with clipnorm = 0.001 was applied during training. Gradient clipping ensures that the norm of the gradient does not exceed the specified threshold (here, 0.001), preventing exploding gradients.

The number of training iterations was generally set to 3000. During training, network weights were saved every 100 steps. For simulations using synthetic data, where the ground-truth choice probabilities were known, the Kullback–Leibler (KL) divergence between the true and model-predicted choice probabilities was computed at each checkpoint. The weights corresponding to the minimum KL divergence were selected, and the RNN with those weights was used as the final model.

For real-world data, we selected the model weights that yielded the lowest cross-entropy loss on the test set, and used the corresponding model as the final output.

Ideally, a separate validation dataset, independent of both the training and test datasets, should be used to determine the optimal number of training iterations (Eckstein et al., 2024). However, given the limited number of subjects in the datasets, we adopted this simplified approach. As a result, we used the models trained for 200 iterations for the Sugawara dataset, 300 iterations for the Palminteri dataset, and 1200 iterations for the Waltmann dataset, corresponding to the training step at which the lowest loss based on the test data was achieved.

### A.6 Performance Metrics

When the ground-truth choice probabilities are available (i.e., when synthetic data are used), the model’s fit can be evaluated by measuring the distance between the model’s predicted choice probabilities and the true probabilities. First, we denoted the choice at trial *t* of subject *s* as $a_t^s$. To quantify the difference between the choice probabilities of the true model $P_{\text {true}}(a_t^s)$ (generated by RL models) and the predictions from the RNN model $P_{\text {pred}}(a_t^s)$, we computed the KL divergence. Given that the probability distributions in our case are Bernoulli distributions (i.e., binary choices), we calculated the KL divergence for $a_t^s$ as follows:

25 $$\begin{aligned} \text {KL}(P_{\text {true}}(a_t^s)||P_{\text {pred}}(a_t^s)) =&P_{\text {true}}(a_t^s = A) \log \left( \frac{P_{\text {true}}(a_t^s = A) + \epsilon }{P_{\text {pred}}(a_t^s = A) + \epsilon } \right) \nonumber \\&+ (1 - P_{\text {true}}(a_t^s = A)) \log \left( \frac{1 - P_{\text {true}}(a_t^s = A) + \epsilon }{1 - P_{\text {pred}}(a_t^s = A) + \epsilon } \right) , \end{aligned}$$

where $\epsilon$ is a small constant (set to $10^{-10}$) added to avoid division by zero or taking the logarithm of zero. The KL divergence is averaged across all subjects and trials. To reduce the computation time, KL divergence was computed once every 10 training iterations.

In real-world behavioral data analysis, the ground truth is unknown. In such cases, model performance cannot be evaluated using metrics such as KL divergence. Instead, it is commonly assessed based on predictive accuracy on held-out test data. Specifically, we computed the normalized log-likelihood (Ito & Doya, 2009) on the test data, or trialwise prediction accuracy (Eckstein et al., 2024), defined as follows:

26 $$\begin{aligned} \text {Normalized likelihood for subject } s = \exp \left( \frac{1}{T_s} \sum _{t=1}^{T_s} \log (P_{\text {pred}}(a_t^s))\right) \end{aligned}$$

Here, $T_s$ denotes the number of trials in the test set for subject *s*.

We performed paired t-tests using the normalized log-likelihoods to compare the predictive performance of the RNN and each RL model. If the RL model showed significantly lower predictive accuracy than the RNN did at the 5% significance level, we considered that the RL model had room for improvement. Since our goal was not to control for familywise error but to evaluate each model individually as a benchmark comparison, we did not apply any correction for multiple comparisons.

### A.7 On-Policy IDT Check

To examine whether a trained RNN has acquired the IDT property, we introduced the *on-policy IDT check*, which involves simulating the RNN as an agent to generate choice data (a process referred to as on-policy simulation or closed-loop simulation), fitting a cognitive model to the generated data, and examining the distribution of estimated parameters.

Specifically, we first defined the RL model parameters for 100 hypothetical agents, ensuring that these parameters span a broad range of plausible values for each parameter: $\alpha$ parameters and $\tau$ are sampled uniformly from [0.1, 0.9], $\beta$ from [1.0, 4.0], and $\varphi$ from [$-$2.5, 2.5]. When multiple candidate RL models can be considered, we selected the one with the highest predictive accuracy for use in this procedure. Each agent performed a two-armed bandit task for 50 trials, generating 100 sessions of behavioral data. These data were then used to update the RNN states for each session through off-policy simulation, allowing the RNN to encode individual characteristics as latent variables.

Next, we used each session-specific RNN state to simulate 5000 trials of a two-armed bandit task in an on-policy manner, where choices were sampled according to the model’s predicted choice probabilities. If the RNN successfully encoded individual differences via latent variables and maintains them stably, the generated 5000-trial data for each session should reflect the corresponding agent’s characteristics.

To evaluate this, we fit an RL model to each session’s 5000-trial data using maximum likelihood estimation. Since the initial 50 trials in the off-policy simulation may introduce biases in early choices—specifically, differences in the initial *Q*-values between options—we accounted for this by allowing the initial *Q*-values to be free parameters in the fitting procedure. Finally, we examined the estimated parameter distributions (e.g., learning rates) across the 100 sessions using histograms. If the recovered distribution covered a wide parameter range, we inferred that the RNN has acquired IDT. Conversely, if the estimated parameters were narrowly concentrated around a single value, we concluded that IDT was not acquired.

### A.8 Real-Word Data

#### Sugawara data and Palminteri Datasets

In the experiments by Sugawara and Katahira (2021) and Palminteri et al. (2017), two feedback conditions were included: a factual condition, where participants were only shown the outcome (rewarded or not rewarded) of the chosen option, and a counterfactual condition, where they also received feedback on the unchosen option. Here, we focused exclusively on the factual condition, which aligns with the paradigms considered in the previous sections.

In both conditions, trials from eight contexts (i.e., stimulus pairs) were presented in an intermixed manner, with four contexts appearing in the first session and the remaining four in the second session. Each session included one “Symmetric,” one “Reversal,” and two “Asymmetric” contexts, resulting in a total of eight stimulus pairs across the two sessions.

Each context was associated with one of three reward probability conditions: In the Symmetric condition, both options had a reward probability of 50%. In the Asymmetric condition, one option had a reward probability of 75% and the other 25%. In the Reversal condition, one option had a reward probability of 83% and the other 17% for the first 12 trials, after which the reward probabilities were reversed for the final 12 trials. Each stimulus pair (context) was presented across 24 trials, resulting in a total of 96 trials per session and 192 trials across the two sessions.

By splitting the dataset into two sets of four contexts, we were able to perform cross-validation at the session (context) level. Specifically, Contexts 1 and 3 (first session) and 6 and 8 (second session) were assigned as training data, whereas Contexts 2 and 4 (first session) and 5 and 7 (second session) were used as test data. This assignment ensured that each of the training and test sets included an equal number of contexts from each of the three reward probability conditions.

The Sugawara dataset included 143 participants (58 females; mean age = 38.7 years, SD = 9.6), and the experiment was conducted online. In contrast, the Palminteri dataset consisted of 20 participants (mean age = 23.9, SD = 0.7), and the experiment was conducted in a laboratory. Unlike the original experiment by Palminteri et al. (2017), Sugawara and Katahira (2021) imposed a 1500 ms response time limit. Trials with missed responses were excluded, and the remaining trials were concatenated for analysis. Among the 143 participants, 10 had at least one missed response, with the number of misses ranging from 4 to 12 (mean $= 5.5$), Thus, a total of 55 trials were missed across all participants.

#### Waltmann Dataset

In a study by Waltmann et al. (2022), 40 participants (20 males; age $= 26.45 \pm 3.88$) completed a probabilistic reversal learning task, which is one specific version of the two-armed bandit task, across two sessions, approximately 1 week apart. All the sessions were conducted in a laboratory setting. Each session consisted of 160 trials in a two-armed bandit setting.

In the first 55 trials of each session, one option was associated with a high reward probability (80%), and the other was associated with a low probability (20%). After trial 55, reward contingencies reversed five times (at trials 55, 70, 90, 105, and 125).

In the present study, we treated the first session as the training data and the second session as the test data. While the second session may be affected by practice effects (Karvelis et al., 2023), we did not attempt to control for or model such effects here.

## Appendix B: Comparison of RNN Architectures

Here, we investigated whether the IDT property, which was demonstrated with the GRU model in the main text, can also be observed in other RNN architectures commonly used as models for human and animal RL, such as the LSTM and the vanilla RNN models. LSTM models, like GRU models, are designed to retain information from distant past histories more effectively, so we expected to see similar behavior in terms of the IDT property. On the other hand, the vanilla RNN simply applies a nonlinear and monotonic $\tanh$ function to the latent units of the linear RNN. It is not obvious whether the IDT property would emerge in the vanilla RNN.

Since the learning speed might differ between RNN architectures, we plotted the KL divergence between the true choice probability and the model’s prediction as a function of the number of training iterations. We also examined the sensitivity to initial conditions by generating multiple learning curves from eight randomly initialized network weights while using the same dataset. The number of iterations was set to 2000 for all the RNN models except for the vanilla RNN. For the vanilla RNN, which exhibited significantly slower convergence, training was repeated for 8000 iterations, and the results were plotted across the full range of iterations. The number of latent units in all the RNNs was set to 10.

Figure 10 shows the results for the same data in Fig. 1 (the ground-truth model is the FQ-learning model with an $\alpha$ value of either 0.1 or 0.9). The KL divergence for the linear RNN converged to approximately 0.05, which we interpreted as the lower bound in the non-IDT mode. For the GRU, the KL divergence ultimately converged toward the IDT mode, approaching 0. However, it exhibits a phenomenon where the KL divergence temporarily remains at approximately 0.05—indicating that the model remains in a non-IDT mode—before suddenly dropping into the IDT mode. This behavior, commonly observed in multilayer neural networks, is referred to as the plateau phenomenon (Fukumizu & Amari, 2000).

For the LSTM model, the near KL = 0 region was reached, indicating the IDT mode, and a plateau phenomenon was observed. However, the convergence speed was relatively slower than that of the GRU, and the degree of dependency on the initial weights was stronger than that of the GRU. Additionally, even after convergence, instability was observed, with multiple temporary spikes where the KL divergence increased.

The vanilla RNN requires much more time to converge. Notably, only the vanilla RNN panel extended up to 8000 iterations, exceeding the number of iterations of the other models. Eventually, the KL divergence approached the region near 0. This finding suggests that even the vanilla RNN is able to reach the IDT mode, but doing so requires many iterations.

Figure 11 shows how the number of GRU units affects the learning curves of an RNN. When the model contained only a single GRU unit, the KL divergence remained around 0.05, corresponding to the upper bound of the no-IDT mode. As the number of units increased, the KL divergence decreased below this threshold, indicating that the model began to acquire the IDT property. Notably, models with more GRU units tended to converge more quickly, regardless of initialization. This outcome is likely because a larger number of weights increases the probability of randomly initializing a configuration that is close to an optimal solution, consistent with the “lottery ticket hypothesis” (Frankle & Carbin, 2018). Although near-optimal solutions were achieved with approximately five units, models with approximately ten units converged more reliably and rapidly. Since increasing the number of units beyond ten yields minimal additional benefit, we used ten GRU units as the default configuration in the main analyses.

## Appendix C: Equivalence Between the FQ-learning and Linear RNN Models

Here, we demonstrate that the FQ-learning and linear RNN models can be equivalent in terms of their input–output relationships by showing the correspondence between their respective parameters.

First, from Eq. (3), the choice probability in FQ-learning is expressed via the difference between the two *Q*-values, $\Delta Q_t = Q_t(A) - Q_t(B)$, as follows:

27 $$\begin{aligned} P(a_t = A)&= \frac{1}{1 + \exp \left( -\beta \Delta Q_t \right) }. \end{aligned}$$

The update rule for the FQ-learning model (Eqs. (1) and (2)) can be rewritten as follows:

28 $$\begin{aligned} Q_{t}(A)&= Q_{t-1}(A) + \alpha (I(a_{t-1}=A) r_{t-1} - Q_{t-1}(A)), \end{aligned}$$

29 $$\begin{aligned} Q_{t}(B)&= Q_{t-1}(B) + \alpha (I(a_{t-1}=B) r_{t-1} - Q_{t-1}(B)). \end{aligned}$$

Here, the indicator function $I(\cdot )$ is 1 when the expression inside is true and 0 otherwise. By subtracting the terms in Eq. (29) from Eq. (28), we obtain

30 $$\begin{aligned} \Delta Q_t \!&=\! (1 - \alpha ) \Delta Q_{t-1} + \alpha (I(a_{t-1}\!=\!1) - I(a_{t-1} \!=\!2)) r_{t-1}. \end{aligned}$$

Now, we consider a linear RNN with a single latent unit ($N_h = 1$). In this case, $\textbf{h}_t$ becomes a scalar, which we denote $h_t$. We demonstrate that $h_t$ can correspond to the variable $\Delta Q_t$, with appropriate weights assigned to the linear RNN. If we assume that $\textbf{b}_x = \textbf{0}$, $\textbf{b}_h = \textbf{0}$, we can write the equations as follows:

31 $$\begin{aligned} h_t&= \textbf{W}_{h} \textbf{x}_t + u_h h_t \end{aligned}$$

32 $$\begin{aligned} \textbf{y}_t&= \textbf{W}_{y} h_t \end{aligned}$$

The input (for choice-dependent reward coding) can be expressed as follows:

33 $$\begin{aligned} \textbf{x}_t = \begin{bmatrix} \tilde{a}_{t-1,1} \\ \tilde{a}_{t-1,2} \\ \tilde{r}_{t-1,1} \\ \tilde{r}_{t-1,2} \end{bmatrix} = \begin{bmatrix} I(a_{t-1} = A) \\ I(a_{t-1} = B) \\ I(a_{t-1} = A) r_{t-1} \\ I(a_{t-1}= B) r_{t-1} \end{bmatrix}. \end{aligned}$$

We further assume that the weight matrix $\textbf{W}_{h}$ has nonzero values only for the components corresponding to $\tilde{r}_t$, specifically set to $\alpha$, $-\alpha$ for the respective options:

34 $$\begin{aligned} \textbf{W}_{h} = \begin{bmatrix} 0&0&\alpha&-\alpha \end{bmatrix}. \end{aligned}$$

Suppose that $u_h = 1-\alpha$; then, Eq. (31) becomes

35 $$\begin{aligned} h_t&= (1 - \alpha ) h_{t-1} + \alpha (I(a_{t-1}=A) - I(a_{t-1} =B)) r_{t-1}. \end{aligned}$$

This formulation is equivalent to replacing $\Delta Q_t$ with $h_t$ in Eq. (30).

If the weight for the output layer is $\textbf{W}_{y} = [\beta , 0]^T$ (where $\cdot ^T$ denotes the transpose), $y_{t,1} - y_{t, 2}$ in Eq. (23) becomes $\beta h_t$, confirming that this linear RNN yields an equivalent output (choice probability) with the FQ-learning model; if the initial value of $h_t$, $h_1$ is the same as that of $\Delta Q_t$ (this is the case if $h_1 = 0$ and $Q_1(A) = Q_1(B)$).

There is ambiguity in the weights of the linear RNN, and there are infinitely many models that can be equivalent to the FQ-learning model. For example, models with $\textbf{W}_{y} = [0.5\beta , -0.5\beta ]^T$ produce the same output. When there are two or more latent units, countless combinations of weights exist that can yield behavior equivalent to that of the linear RNN with a single latent unit.

## Appendix D: RNNs Track Within-subject Parameter Variability

As shown in Fig. 1B, the RNN appears to capture individual differences during the initial trials and subsequently generates behavior that remains consistent with those differences. However, in reality, parameters such as the learning rate may also vary within individuals during the task (e.g., Behrens et al., 2007; Browning et al., 2015). To examine whether the RNN can also track such within-subject fluctuations in parameters, we consider two cases: one in which the ground-truth data used to train the RNN have constant learning rates and another in which the ground-truth learning rate changes during the task.

Figure 12A shows the predictions of an RNN trained based on data from Scenario 1, where the true model is the FQ-learning model and the learning rate ($\alpha$) remains constant throughout the task (either 0.1 or 0.9). In the test data, however, the learning rate switches after 100 trials (from $\alpha =0.1$ to 0.9 or vice versa). For agents whose learning rate changes from 0.1 to 0.9, the RNN fails to track the change in $\alpha$, continuing to produce behavior consistent with a slow learning rate (left panel). In contrast, when $\alpha$ changes from 0.9 to 0.1, the RNN gradually adapts and slows the rate of change in its choice probabilities accordingly (right panel). These results reveal an asymmetry in the RNN’s tracking ability: it can follow transitions from fast to slow learning, but not the reverse, when such transitions are not present during training.

Figure 12B shows the results when the RNN is trained based on data that include the learning rate shift (from $\alpha =0.1$ to 0.9 and vice versa). In this case, the RNN successfully tracks the changes in both directions, demonstrating that RNNs can learn to adapt to within-subject changes in parameters when such patterns are present in the training data.

## Footnotes

1 When there is no state variable as in the present study assumed, *Q*-learning and other variants of the RL algorithm, Sarsa, are indistinguishable (Sutton & Barto, 2018); however, following convention, we refer to it as Q-learning. This model is also referred to as the Rescorla-Wagner model or delta rule.

## Code Availability

All code used for the simulations, analyses, and figures is available at https://osf.io/hnb7q/.
