# Supplementary material for: Excessive Flexibility? Recurrent Neural Networks Can Accommodate Individual Differences in Reinforcement Learning Through In-Context Adaptation
Source: Comput Brain Behav. 2025 Jul 18;9(1):34–61. doi: 10.1007/s42113-025-00254-8 (PMC13262387; doi:10.1007/s42113-025-00254-8)
Supplement: Supplementary file 1 — (pdf 1255 KB) [file 42113_2025_254_MOESM1_ESM.pdf]

Supplementary Information for Excessive flexibility? Recurrent neural networks can accommodate individual differences in reinforcement learning through in-context adaptation

Kentaro Katahira

Human Informatics and Interaction Research Institute, National Institute of Advanced Industrial Science and Technology (AIST), Tsukuba, Japan

E-mail: k.katahira@aist.go.jp

Supplementary Figures

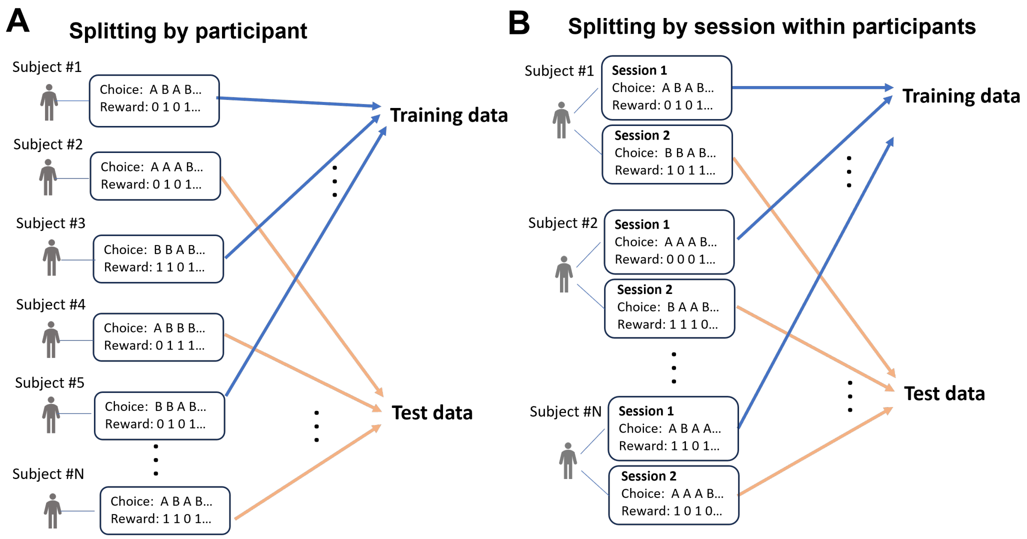

Fig. S1. Schematic diagram of two approaches for splitting data into training and test sets. (A) Splitting by participant: The data are split at the participant level, with some participants allocated for training and others for testing. (B) Splitting by session within participants: When participants complete multiple sessions (e.g., using different stimulus pairs), the data can be split at the session level, with some sessions used for training and others for testing.

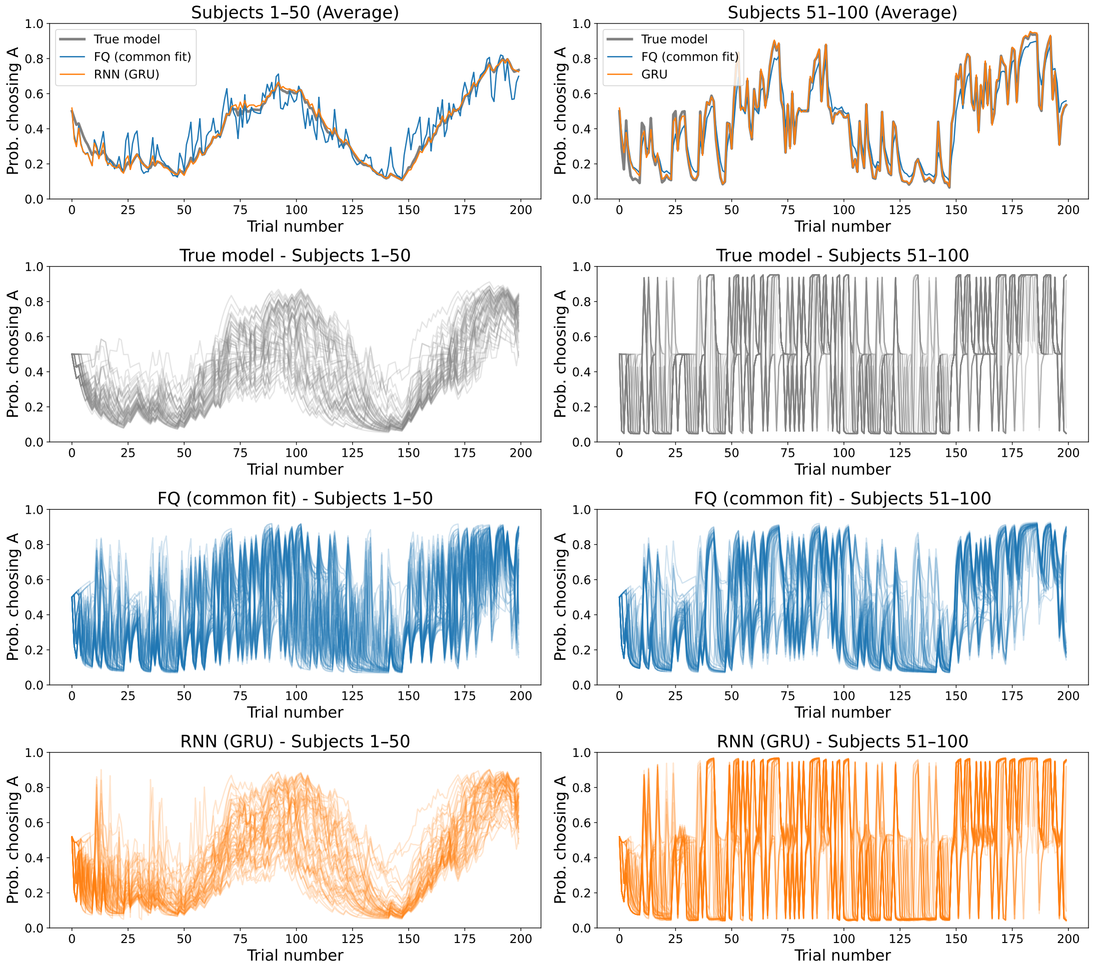

Fig. S2. Choice probability trajectories corresponding to the results in Fig. 1B (Scenario 1). The first row shows the average choice probabilities for each model, computed across all the subjects within each group. The left and right columns correspond to the low learning rate group ($\alpha = 0.1$) and the high learning rate group ($\alpha = 0.9$), respectively. The gray line represents the true choice probabilities generated by the ground-truth model (FQ-learning), whereas the blue and orange lines represent the predictions from the RL model (common fit) and the trained RNN (GRU), respectively. The second row displays the trial-by-trial choice probabilities of individual subjects under the true model, whereas the third and fourth rows show the corresponding predictions from the RL model and GRU, respectively.

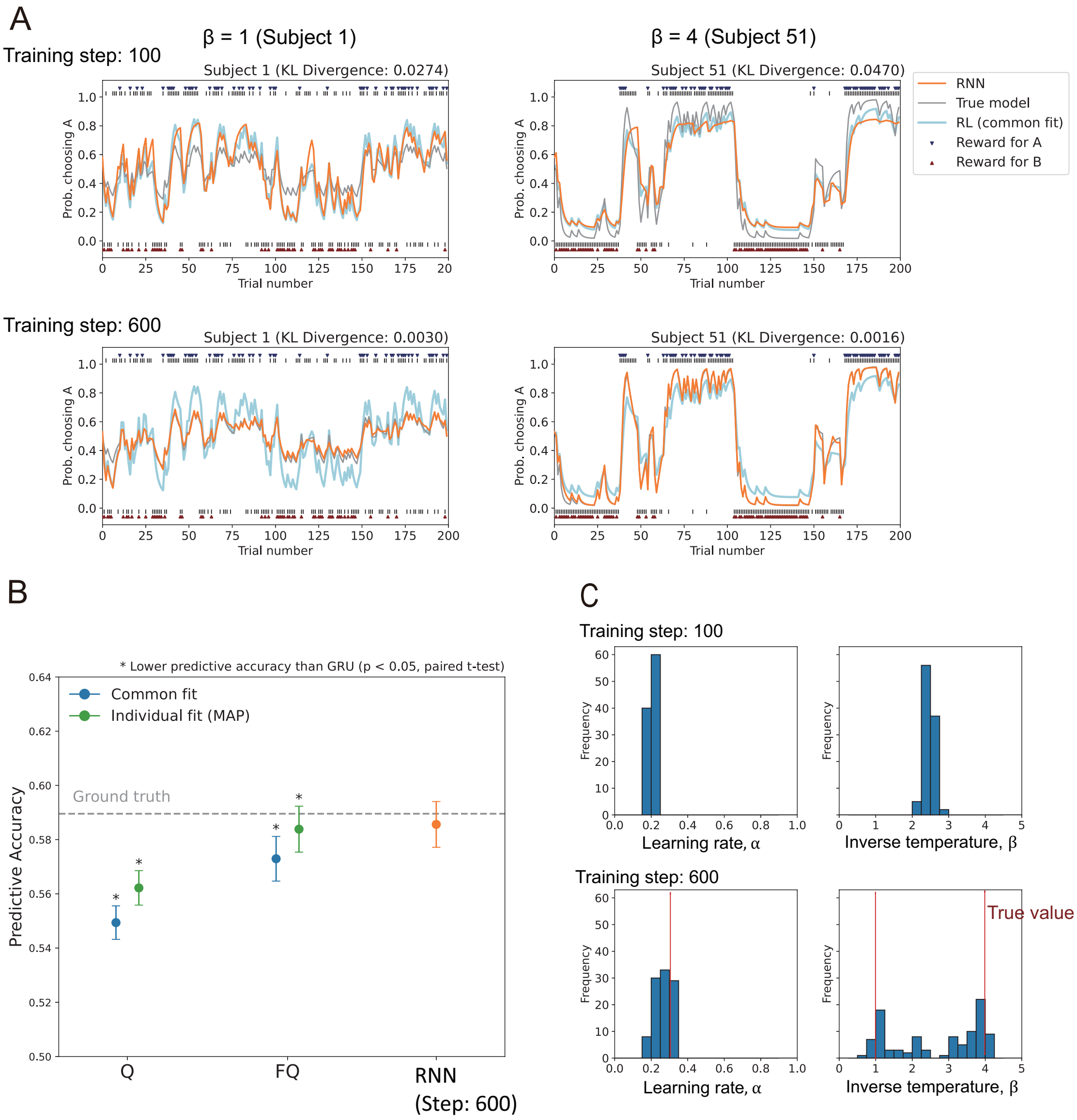

Fig. S3. Simulation results where the ground-truth model is the FQ learning model and individual differences exist in the inverse temperature parameter β. Subjects 1–50 belong to a low-β group ($\beta = 1.0$), and subjects 51-100 belong to a high-β group ($\beta = 4.0$). (A) Predicted choice probabilities from the RNN at training steps 100 and 600, the common-fit FQ-learning model, and the ground-truth model. (B) Predictive accuracy on the test data. The RNN model used the weights from 600 training steps, yielding the lowest KL divergence. (C) Results of the on-policy IDT check for RNNs at training steps 100 and 600. Distributions of parameter estimated are shown for the FQ-learning model fit to choice data simulated from the RNN are shown. The conventions follow those of Fig. 5 in the main text.

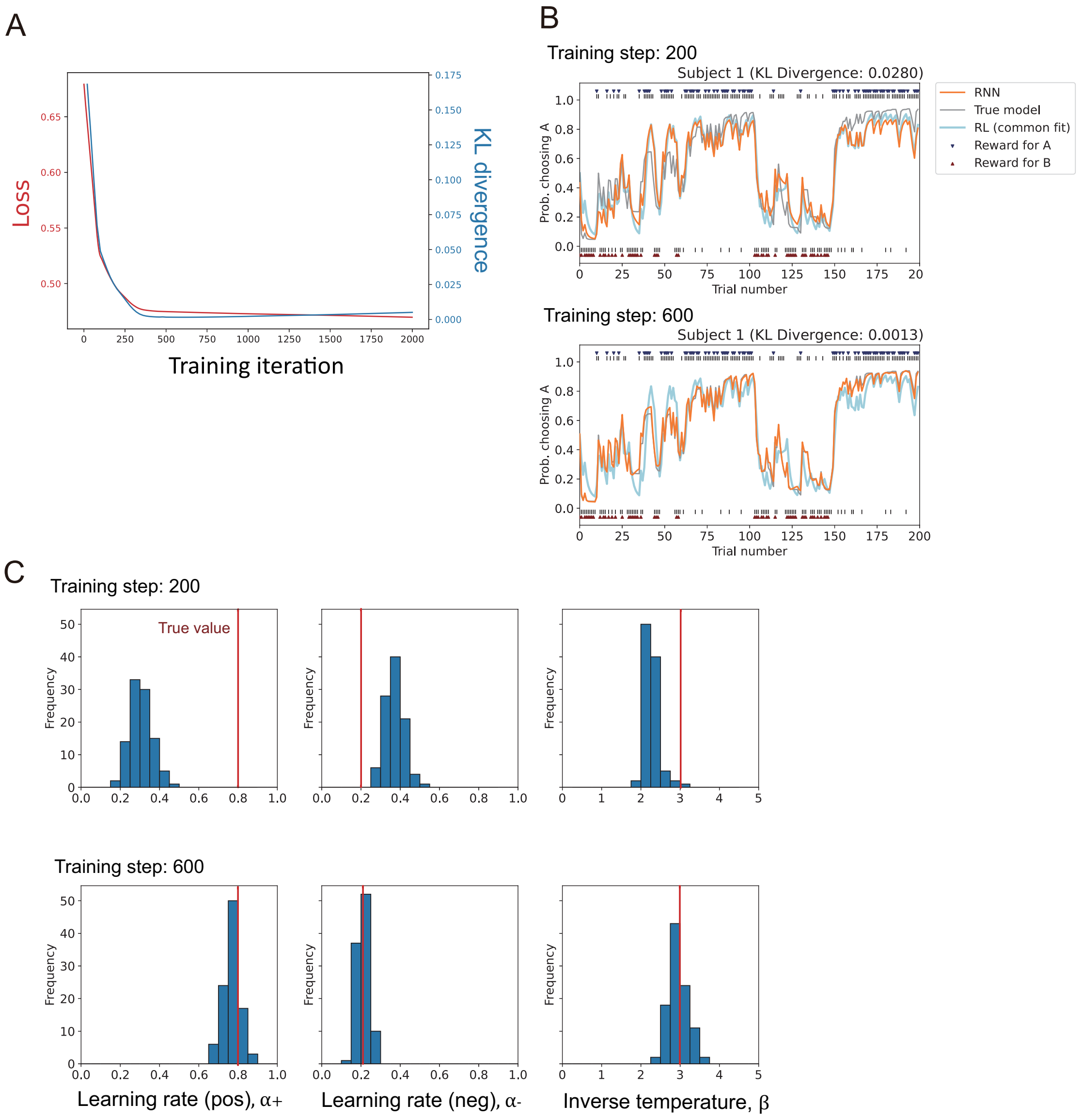

Fig. S4. RNN behavior and on-policy IDT check when the Q+A model without individual differences is the ground truth (Scenario 2b). (A) Learning curve of the RNN, showing the loss and KL divergence during training. (B) Time series of choice probabilities predicted by the RNN at training iterations 200 and 600. The model at 600 iterations corresponds to the point at which KL divergence is minimized. (C) Results of the on-policy IDT check: parameter estimates from fitting the Q+A model to simulated data generated by the RNN trained at 200 and 600 iterations. The red vertical lines indicate the true parameter values used to generate the data.

Supplementary Text

S1 Sensitivity of IDT to the Strength of Individual Differences

Here, we examine how the IDT property of an RNN affects the model's predictive accuracy, depending on the degree of individual differences. In the simulation for generating data, the learning rate for the low-learning-rate group was set to $\alpha - 0.5\Delta\alpha$, while that for the high-learning-rate group was set to $\alpha + 0.5\Delta\alpha$. The value of $\Delta\alpha$ was varied from 0 to 0.9 in increments of 0.1. The inverse temperature β was fixed at 3.0 for all agents. The test samples used for cross-validation follow the same distribution of learning rates, generating data for 100 subjects independently from the training data. The normalized likelihood for the test data (the probability for actual selected choice per trial) served as the metric for predictive accuracy (see the Appendix "Performance metrics" in the main text).

Figure S5 shows the normalized likelihood of the RL and RNN models, along with that of the ground-truth model (FQ-learning), for both training and test data. The solid gray line represents the likelihood of the ground-truth and serves as an upper bound that no fitted model should exceed. As $\Delta\alpha$ increases and individual heterogeneity becomes stronger, this upper bound tends to decrease, which is likely due to increased randomness in the choices of agents with smaller α values.

Let us first consider the individually fitted FQ-learning model (blue line). For the training data (Panel A), this model achieves higher likelihood than the true model does, likely reflecting overfitting, as the normalized likelihood (i.e., predictive accuracy) on the test data falls below that of the true model. Notably, in the region where individual differences are small ($\Delta\alpha \leq 0.2$), the predictive performance of the individual-fit model is even lower than that of the common-fit FQ-learning model. This outcome likely reflects the fact that the common-fit model is less affected by estimation noise and is less prone to overfitting.

The RNN (orange line) outperforms the common-fit FQ-learning model using both training and test data when $\Delta\alpha \geq 0.4$. This improvement can be attributed to the RNN's ability to capture individual differences via IDT. However, even with the test data, the RNN performs worse than the individually fitted FQ-learning model does, indicating limitations in the RNN's ability to fully capture individual-level variability.

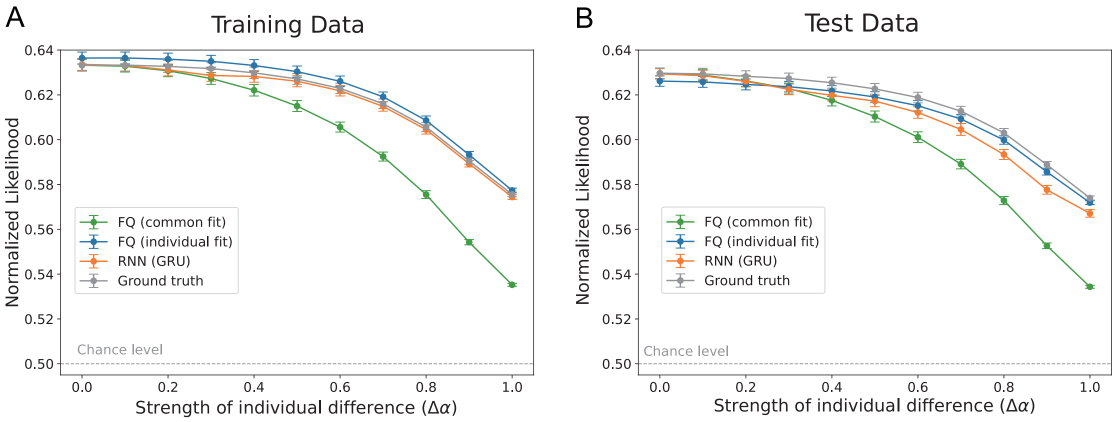

Fig. S5. Normalized log-likelihood per trial as a function of individual difference strength $\Delta\alpha$. (A) Results for the training data. (B) Results for the test data. The (true) learning rates were set as $\alpha = 0.5 - \Delta\alpha$ for the low-learning-rate subjects and $\alpha = 0.5 + \Delta\alpha$ for the high-learning-rate subjects. The gray line represents the normalized log-likelihood of the FQ-learning model used to generate the data (i.e., the ground-truth model), which serves as a theoretical upper bound. The green line shows the performance of the common-fit FQ-learning model, the blue line represents the individual-fit FQ-learning model, and the orange line corresponds to the RNN (GRU). Each line indicates the average over 50 runs, and error bars denote the standard error of the mean.

S2 Behavior of the Latent Units in the GRU

In Figure S6, we plot the time series of the latent units $\mathbf{h}_t$ of the RNN, where the number of GRU units is set to six for better visualization. While principal component analysis (PCA) has often been used for visualizing the behavior of latent units (e.g., Eckstein, Summerfield, Daw, & Miller, 2024), we chose to plot the raw unit values instead, as the six latent units still demonstrate comparable IDT capabilities. In the left panels, where the true learning rate is low, the value of Unit #4 (red, indicated by the vertical arrow) remains high after the first 30 trials. Conversely, in the right panel, for high learning rate subjects, the behavior of this unit tends to stay mostly below zero; although it gradually increases with the selection of option B, it then sharply decreases to a low value. These behaviors of Unit #4 are thought to be involved in adjusting and maintaining the effective learning rate.

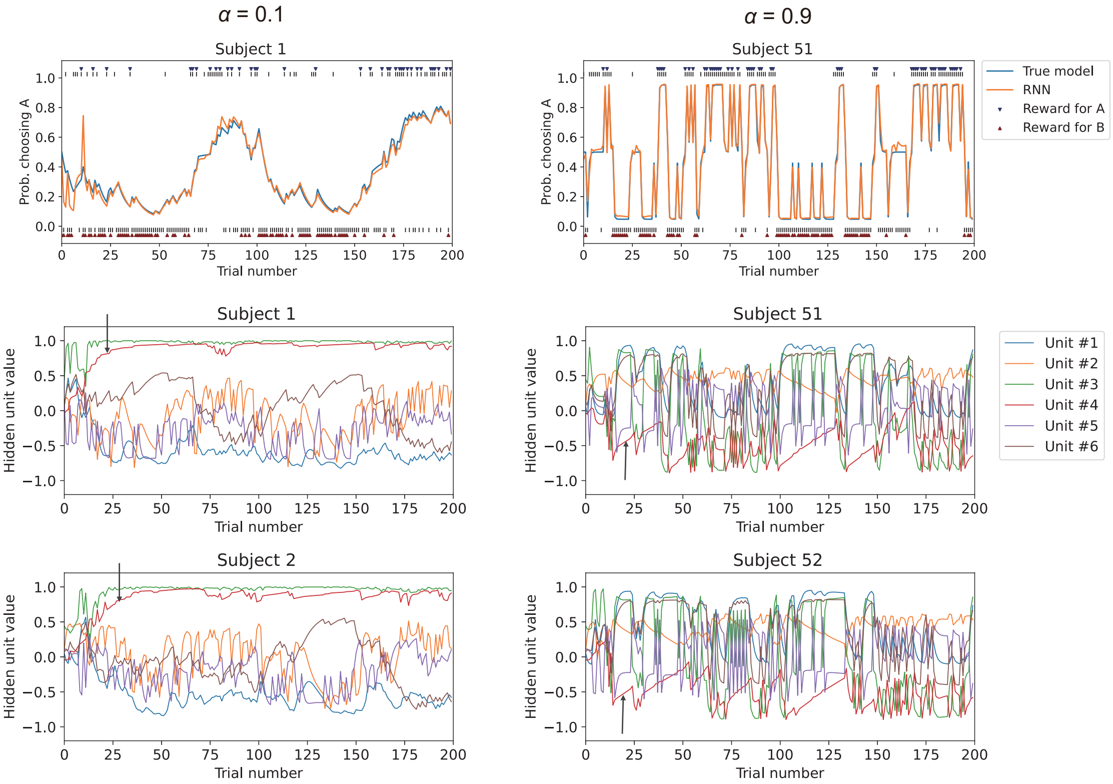

Fig. S6. Behavior of the latent units in the GRU. The upper panel corresponds to Fig. 1 of the main text, except that the number of GRU units is set to six for better visualization of the latent units. The middle row shows the behavior of the GRU's latent units for a virtual subject corresponding to the upper panel. The lower row provides examples from other subjects. The arrow indicates Unit #4, a representative unit that is thought to reflect the effective learning rate.

References

Eckstein, M. K., Summerfield, C., Daw, N., & Miller, K. J. (2024). Hybrid neural-cognitive models reveal how memory shapes human reward learning. *PsyArXiv*.
